# Opposing roles for GSK3β and ERK1-dependent phosphorylation of huntingtin during neuronal dysfunction and cell death in Huntington’s disease

**DOI:** 10.1038/s41419-025-07524-0

**Published:** 2025-04-22

**Authors:** Thomas J. Krzystek, Rasika Rathnayake, Jia Zeng, Jing Huang, Gary Iacobucci, Michael C. Yu, Shermali Gunawardena

**Affiliations:** 1 Department of Biological Sciences, The State University of New York at Buffalo, Buffalo, NY USA; 2 Neuroscience Program, The State University of New York at Buffalo, Buffalo, NY USA

**Keywords:** Huntington's disease, Mechanisms of disease

## Abstract

Huntington’s disease (HD) is a devastating neurodegenerative disorder that manifests from an N-terminal polyQ-expansion (>35) in the Huntingtin (*HTT*) gene leading to axonal degeneration and significant neuronal death. Despite evidence for a scaffolding role for HTT in membrane-related processes such as endocytosis, vesicle transport, and vesicle fusion, it remains unclear how polyQ-expansion alters membrane binding during these processes. Using quantitative Mass Spectrometry-based proteomics on HTT-containing light vesicle membranes isolated from healthy and HD iPSC-derived neurons, we found significant changes in the proteome and kinome of signal transduction, neuronal translation, trafficking, and axon guidance-related processes. Through a combination of in vitro kinase assays, *Drosophila* genetics, and pharmacological inhibitors, we identified that GSK3β and ERK1 phosphorylate HTT and that these events play distinct and opposing roles during HD with inhibition of GSK3β decreasing polyQ-mediated axonal transport defects and neuronal cell death, while inhibition of ERK enhancing these phenotypes. Together, this work proposes two novel pathways in which GSK3β phosphorylation events exacerbate and ERK phosphorylation events mitigate HD-dependent neuronal dysfunction highlighting a highly druggable pathway for targeted therapeutics using already available small molecules.

## Highlights

- The proteome and kinome of vesicular/light membranes are dysregulated in HD.
- HTT phosphorylation levels are elevated in HD.
- GSK3β phosphorylates HTT, contributing to HTT and global axonal transport defects.
- ERK1 phosphorylates HTT and is neuroprotective against HD-mediated neuronal death.
- GSK3β phosphorylation events exacerbate while ERK phosphorylation events mitigate HD-dependent neuronal dysfunction

## Introduction

Huntington’s disease (HD) is a neurodegenerative disorder that manifests from an expansion of CAG repeats (>36) in the Huntingtin (*HTT*) gene. Although identified more than two decades ago, the normal function of HTT remains elusive despite being ubiquitously expressed [1], enriched within neurons [1, 2], and essential for embryonic development [3]. One proposed function is a role as a scaffolding protein which is supported by the broad distribution of N- and C-terminal HEAT (Huntingtin, Elongator factor3, PR65/A regulatory subunit of PP2A, and Tor1) repeats thought to enhance protein-protein interactions [4–6]. Indeed, early yeast two-hybrid ([7, 8]) or immunoprecipitation-mass spectrometry (MS; [9, 10]) studies identified >350 HTT-binding partners linked to gene expression, metabolism, proteostasis, endocytosis, and trafficking [11]. Although polyQ-expansion can dramatically alter the HTT interactome [9, 12], there is no clear insight yet into the normal functions of HTT with these binding partners, thus hindering our current understanding of pathways involved in the induction of neuronal pathogenesis in HD.

Despite lacking a transmembrane domain, HTT can localize to membranes [13] and can associate with phospholipids [14–16], likely through a putative N-terminal amphipathic α-helical domain in the first 17/18 amino acids of HTT [14, 17–20]. In fact, HTT localizes across a broad range of different membranous structures: vesicles [13, 21], plasma membrane [14], endoplasmic reticulum, Golgi, and endosomes [17, 22–24]. Although work has shown that HTT’s association with membranes is altered with polyQ-expansion [12, 14, 15, 25, 26], little is known about the interacting partners at these membranes, hampering our ability to identify candidate targets for therapeutic interventions aimed at defective pathways such as neural adhesion [27], vesicle fusion [28], endocytosis [29], and axonal transport of synaptic [30, 31] and Rab-containing vesicles [32–34]. Therefore, elucidating the proteomic landscape of HTT-associated membranes under normal and diseased states is critical in unraveling the underlying complicated mechanisms of HD pathogenesis.

Here, we address these gaps in knowledge by testing the hypothesis that polyQ-expansion hinders HTT-mediated scaffolding at membranes by enriching HTT-containing light membranes (LMs) from healthy (Q17) and diseased (Q109) iPSC-derived neurons for quantitative MS analysis. We found a dramatic redistribution of kinases at HD LMs, which coincided with increased levels of phosphorylated HTT. Although prior MS-based studies identified dozens of putative phosphorylation consensus sites on HTT [35, 36], the functional relevance of these is yet to be examined. In addition to AKT, which is well characterized in the context of HTT’s normal function and during HD [37, 38], we identified that polyQ-expansion altered the distribution of several kinases: CDK1/5, ERK1/2, and GSK3α/β. Interestingly, the sole putative phosphorylation site for GSK3α/β overlaps with one of three putative sites for ERK1/2, suggesting a potential interplay between GSK3- and ERK-related pathways with normal HTT (Q17) and/or pathogenic (Q109) HTT and in HD. By combining in vitro kinase assays and pharmacological inhibitors, we identified that GSK3β and ERK1 can phosphorylate both normal and pathogenic HTT. In a humanized model of HD in *Drosophila* GSK3β inhibition rescued polyQ-mediated axonal transport defects, synaptic dysfunction, and neuronal cell death. In contrast, ERK inhibition enhanced neuronal cell death and axonal defects, which were rescued by adding excess ERK. Taken together, our observations identify previously unknown discrete and opposing roles for ERK and GSK3β kinases, with ERK playing an antagonist role in pathogenic HTT-mediated cell death and GSK3β exacerbating pathogenic HTT-mediated axonal transport defects. This work highlights a novel pathway for potential therapeutic interventions that can aid to mitigate early (transport deficits), and terminal (cell death) consequences seen in HD.

## Results

### Pathogenic HTT disrupts the kinome network in membranes

**Fig. 1:**
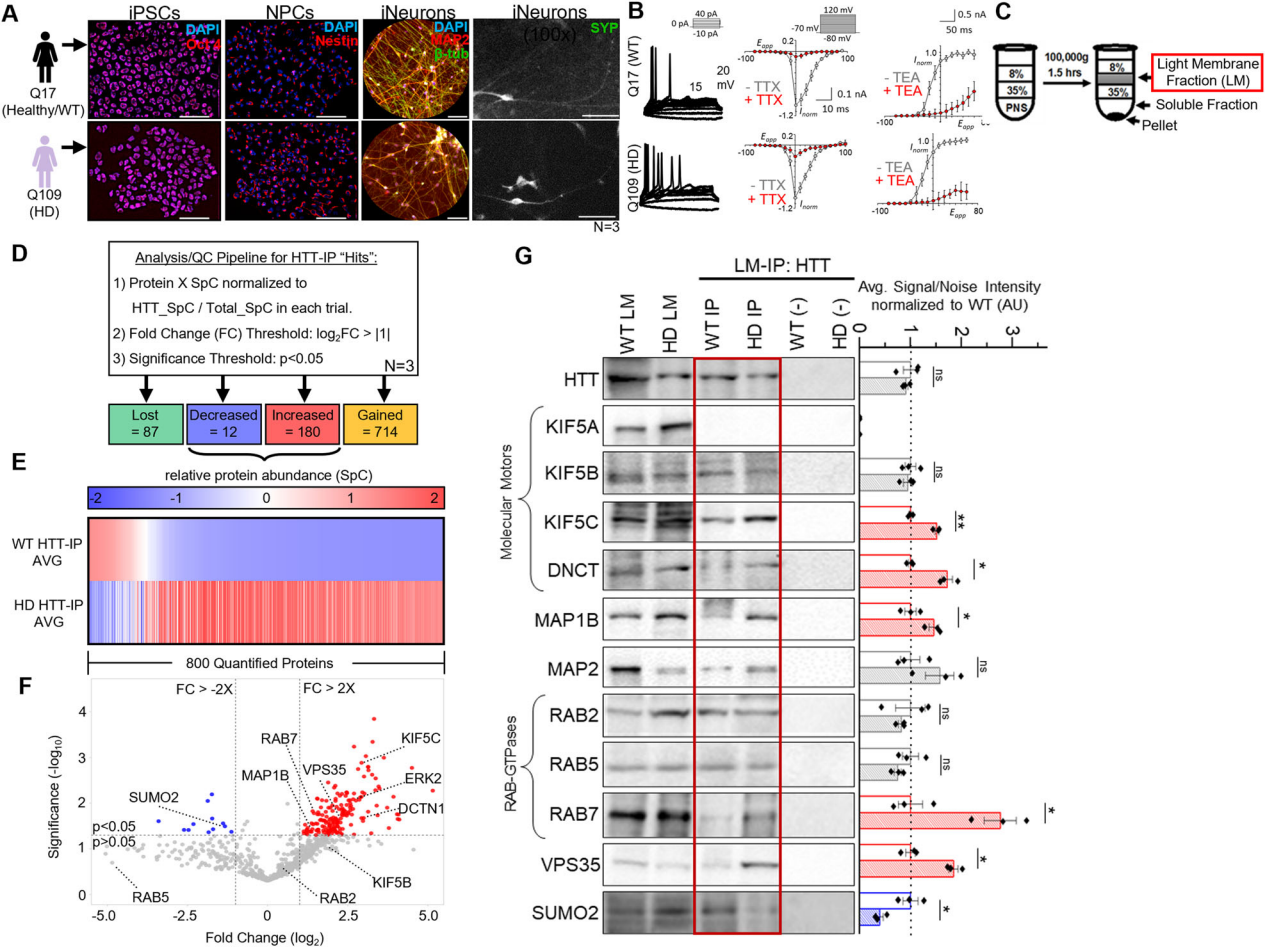
The proteomic network on HTT containing light membranes is dramatically altered in HD iPSC-derived neurons. **A** Representative images from healthy/normal (WT, Q17) or diseased (HD, Q109) human iPSCs stained with the pluripotent marker OCT-4, the neuronal precursor (NPC) marker Nestin and the mature neuronal markers MAP2 and βIII-Tubulin. Hoechst stains nuclei. Scale = 25 μm. Differentiated neurons show Synaptophysin (SYP) positive staining. Scale = 10 μm. **B** Electrophysiological analysis of WT and HD human neurons differentiated from iPSCs show action potentials, which are abolished in the presence of TTX or TEA. **C** Schematic diagram of human iNeuron lysate fractionation into perinuclear supernatant (PNS), light membrane (LM), soluble (SF), and heavy membrane (P1) fractions by ultra-centrifugation and sucrose gradient separation. **D** Workflow for quality control and quantification of unique peptides identified from LC-MS of HTT-IPs from WT or HD human iNeurons. **E** Hierarchical cluster heat map showing the avg. relative abundance (spectral count; SpC) of 800 proteins (≥3 unique peptides/trial across ≥2 biological replicates) quantified across the WT and HD HTT-IPs with a normalized fold change (FC) threshold of ±2X and a significance threshold of *p* < 0.05 determined by a Welch’s *t* test across three independent biological replicates. Increased in HD HTT-IP = red, decreased in HD HTT-IP = blue. In addition, proteins were identified in only WT HTT-IP (lost = green) or in only HD HTT-IP (gained = orange). **F** Volcano plot with the *y* axis depicting significance (−log_10_[*p* value]) and the *x* axis depicting fold change of individual peptides between HD and WT HTT-IPs (log_2_[FC]). Three independent biological replicates were performed for each genotype. A negative, no-antibody IP was performed to account for non-specific peptide association with magnetic beads. **G** Representative western blot of HTT-IP from WT or HD LMs, probed against HTT, KIF5A, KIF5B, KIF5C, DNCT, MAP1B, MAP2, RAB2, RAB5, RAB7, VPS35, or SUMO2. Except for KIF5A, all show presence in WT and HD HTT-IP. No bands are seen in the negative no antibody control (−Crtl). *n* = 3. Statistical analysis was conducted using the two-sample two-sided Student’s *t* test comparing signal/noise intensity between bands in WT and HD conditions normalized to WT. Data represented as mean ± SEM. ns = *p* > 0.05, *p < 0.05, **p < 0.005.

To test the hypothesis that expansion of polyQ repeats alters the scaffolding role of HTT at membranes, we isolated LMs involved in trafficking, regulation of trafficking, and downstream events of trafficking by using terminally differentiated neurons from Q17 (WT) or Q109 (HD) patient-derived iPSCs (iNeurons) (Fig. 1A–C; Fig. S1A; [33, 34]). LMs were first isolated as we have done previously using a subcellular fractionation and membrane flotation on sucrose step gradient protocol where membrane-associated proteins are enriched in the 35/8 sucrose interface membrane fraction with syntaxin-1, synaptotagmin1 (SYT), synaptobrevin (SYB) and Rab proteins, while Golgi, ER, and mitochondrial proteins are enriched in the pellet [34, 39–42]. Previous proteomics analysis has shown that these membrane proteins are involved in signal transduction and membrane trafficking [41].

To evaluate how pathogenic HTT changes the proteomic network of HTT-associated membranes, we immunoprecipitated (IP’d) HTT from WT or HD LMS isolated from iNeurons and then subjected these samples to MS analysis. Our quality control pipeline (Fig. 1D–F) required identified proteins to be detected in at least two biological replicates with the presence of at least three unique peptides (spectral count >3). The proteomic network of the HTT-LMs from HD iNeurons exhibited 894 gained/increased (orange/red) and 99 lost/decreased (green/blue) proteins compared to WT iNeurons (Fig. 1D–F). In contrast, the proteomic network of the total membranes isolated from HD iNeurons exhibited 269 gained/increased (orange/red) and 1,186 lost/decreased (green/blue) proteins compared to WT iNeurons (Fig. S2A–C). Interestingly, HTT showed a decreased presence across total LMs in HD iNeurons, suggesting that HTT’s intracellular localization and/or membrane association is likely altered with pathogenic polyQ-containing HTT. Notably, we isolated previously identified targets capable of interacting with HTT, such as HTT-associated protein 40 (HAP40; [43]), HTT-interacting protein K (HYPK; [44]), HTT-interacting protein 1 (HIP1; [45, 46]), HTT-associated protein 1 (HAP1; [47]), dynein [48], and dynactin [49].

To better understand the biological relevance of the observed HD-mediated proteomic shift, we coupled Gene Ontology (GO) analysis with reactome pathway enrichment analysis using clusters of decreased, increased, lost, or gained proteins from total LMs (Fig. S3A, C, E, G) or HTT LMs (Fig. S3B, D, F, H). We found that pathogenic HTT largely affected protein clusters that show enrichment at synapses, axons/dendrites, the endomembrane system, and synaptic vesicles (Fig. S3A, B, E, F) with involvement in pathways related to translation, trafficking, axon guidance, and intracellular signaling (Fig. S3C, D, G, H). Having previously identified HTT-relevant roles in the trafficking of a sub-type of RAB-containing vesicles [32, 33, 50], we next focused on the validation of our observations. Indeed, we observed RAB7 with non-pathogenic HTT LMs with a significantly increased association seen in pathogenic HTT LMs (Fig. 1G), suggesting a possible accumulation of this complex due to defective transport and/or endolysosomal degradation mechanisms. We further validated significant changes in molecular motor proteins kinesin-1 (KIF5C) and dynactin (DNCT), the microtubule-binding proteins MAP1B and MAP2, the RAB-GTPases RAB2 and RAB5, and other vesicle or trafficking-related proteins such as SYT1, tyrosine hydroxylase (TH), and VPS35 (Fig. 1G and S2D, E) collectively supporting our hypothesis that pathogenic HTT disrupts its scaffolding function on membranes. This disruption consequently led to widespread alterations in proteomic networks involved in trafficking, and other pathways that will need to be further examined (translation, axon guidance, and intracellular signaling) in future studies.

**Fig. 2:**
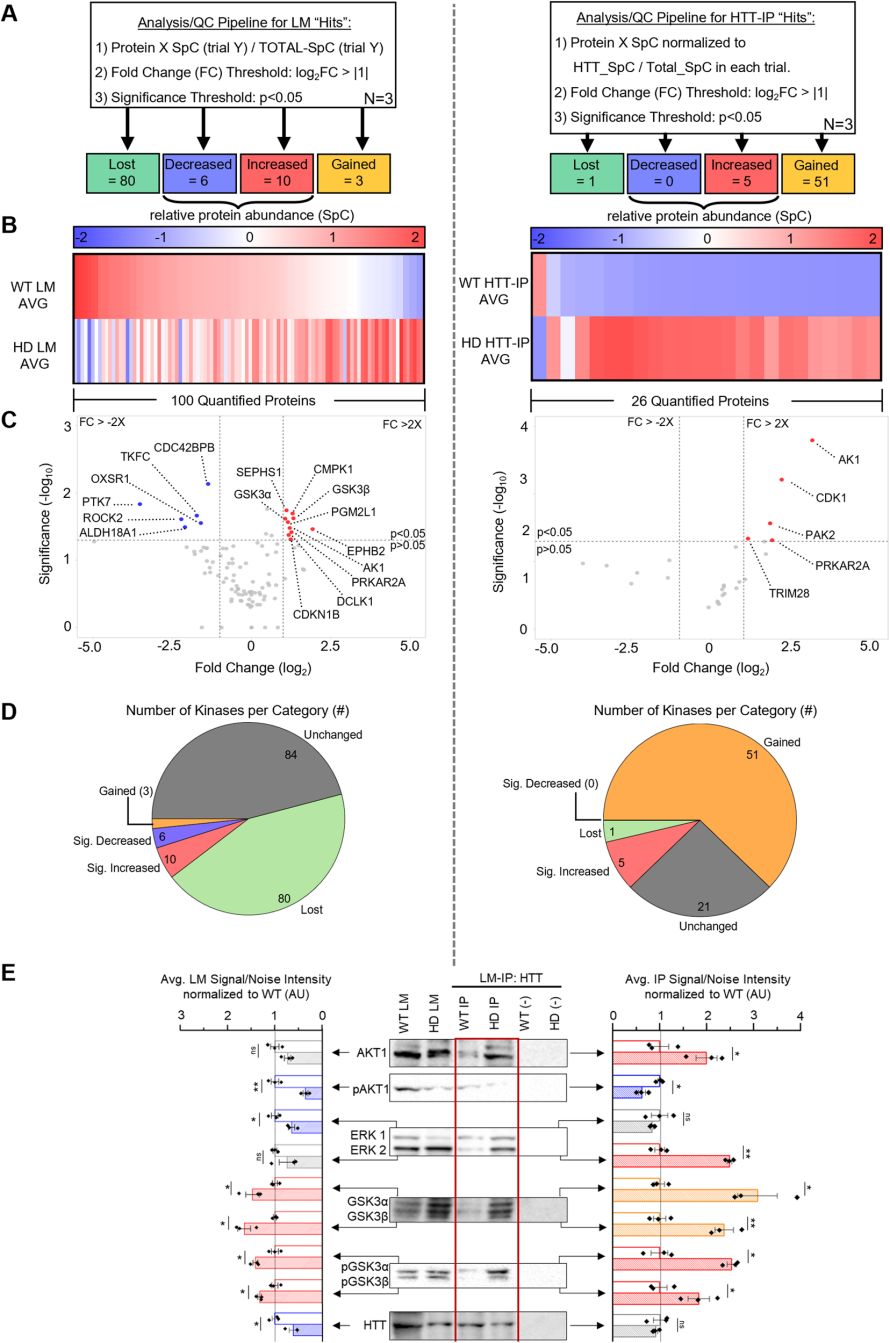
Pathogenic HTT triggers abnormal kinase associations with HTT and on membranes. **A** Workflow for quality control and quantification of unique peptides identified as kinases from LC-MS of total LMs (left) or HTT-IPs (right) from WT or HD iNeurons. **B** Hierarchical cluster heat map showing the avg. relative abundance (spectral count; SpC) of kinases (≥3 unique peptides/trial across ≥2 trials) quantified across total LMs (left) and HTT-IPs (right) from WT and HD iNeurons with a normalized fold change (FC) threshold of ±2X and a significance threshold of *p* < 0.05 determined by a Welch’s *t* test across three independent biological replicates. Increased in HD = red, decreased in HD = blue. In addition, kinases were identified in only WT (lost = green) or in only HD (gained = orange). **C** Volcano plot with the y-axis depicting significance (−log10[*p* value]) and the *x* axis depicting fold change of individual peptides between LMs (left), or HTT-IPs (right) enriched from HD or WT iNeurons (log2[FC]). Three independent biological replicates were performed for genotype. A negative, no-antibody IP was performed to account for non-specific peptide association with magnetic beads. **D** Depiction of the number of kinases in LMs (left) or HTT-IPs (right) that show increased (Red), decreased (blue), lost (Green), gained (orange), or unchanged (grey) abundance between WT and HD iNeurons. **E** Representative western blot with quantification of LMs (left) or HTT-IPs (right) from WT or HD iNeuron samples, probed with AKT1, pAKT (pSer473), ERK1/2, GSK3α/β, or pGSK3α/β (pTyr279/pTyr216), which all show presence in WT and HD HTT-IP. No bands are seen in the negative no antibody control (−Crtl). *n* = 3. Statistical analysis was conducted using the two-sample two-sided Student’s *t* test comparing signal/noise intensity between bands in WT and HD conditions normalized to WT. Data represented as mean ± SEM. ns = *p* > 0.05, *p < 0.05, **p < 0.005.

Dysfunctional kinase signaling pathways involving AKT [37, 51–53], YAP [54], ERK (extracellular signal-related kinase; [55, 56]), mTOR (mechanistic target of rapamycin; [57]), GSK3 (Glycogen synthase kinase 3; [58]), and CDK5 (cyclin-dependent kinase 5; [59, 60]) were proposed to contribute to HD pathogenesis. However, aside from AKT [37], little is known about these kinase-mediated mechanisms. Our reactome pathway enrichment analysis of proteins identified with HTT at membranes showed proteins involved in signal transduction and signaling pathways were collectively among the top-most represented identifiers (Fig. S3C, D, G, H). Therefore, we focused on the human kinome with the prediction that pathogenic HTT alters kinase associations on membranes. Indeed, we observed a dramatic redistribution of kinases associated with HTT on membranes in HD iNeurons (compared to normal/WT), with 56 gained/increased and 1 lost/decreased associations in HD (Fig. 2A–D, right). However, since pathogenic HTT can also exert secondary effects that alter the localization/recruitment of kinases across membranes that may not necessarily contain HTT, we identified 86 lost/decreased and 13 gained/increased kinases across total membranes in HD compared to normal (Fig. 2A–D, left). Furthermore, there were 64 lost/decreased kinases and 5 gained/increased kinases on total membranes that exhibited no association with HTT (Table S1). These findings suggest that pathogenic HTT causes a shift in the localization of these kinases across membranes. This proposal is further supported by 21 kinases showing gained/increased associations with HTT in HD iNeurons compared to normal, and a concomitant lost/decreased association across total membranes (Table S2), implicating that these proteins are likely sequestered with pathogenic HTT.

Since pathogenic HTT led to altered kinase associations across a broad range of kinome families (Fig. S4A), we next performed reactome pathway enrichment analysis on the kinases exhibiting HTT-dependent associations (Fig. S4B) to begin dissecting the biological relevance of these alterations. Notably, we found that pathogenic HTT affected kinases involved in PI3K, ERK, mTOR, MAPK, AKT, GSK3, and TP53 signaling pathways (Fig. S4B). Intriguingly, previous MS-based evidence suggested that several of these kinases can putatively phosphorylate HTT (Table 1, [35]), including AKT1 (Ser421), ERK1/2 (Ser2076, Ser2653, and Ser2657), CDK1/5 (Ser1181 and Ser1201), or GSK3α/β (Ser2657). While only CDK5 [59, 60] and AKT1 [37] have been validated to directly phosphorylate HTT, we predict that the phosphorylation state of HTT at membranes becomes dysregulated in HD. Indeed, in addition to the elevated levels of total GSK3α/β at both HTT-LMs and total LMs in HD iNeurons (Fig. 2C–E, Table 1), we observed a significant increase in the levels of the phospho-active forms of GSK3α/β (pTyr279/pTyr216; [61, 62]), indicating enhanced kinase activity at these membranes (Fig. 2E). In contrast, the levels of total AKT1 and active AKT1 (pSer473; [63]) were decreased at total membranes in HD iNeurons (Fig. 2C–E, Table 1). The fact that AKT1 can directly inhibit GSK3 activity [64] could explain our observation of increased active GSK3 levels concomitant with decreased active AKT1 levels. Furthermore, ERK1 was also decreased at total membranes in HD iNeurons (Fig. 2C–E, Table 1) and has been previously shown to antagonize GSK3-mediated signaling in cortical neurons [65]. Although AKT1-mediated phosphorylation of HTT (Ser421) has been well characterized in the context of scaffolding roles for kinesin-1 and dynein during axonal transport [38], it remains unknown whether ERK1 or GSK3α/β can phosphorylate HTT and how these phosphorylation events contribute to neuronal dysfunction in HD.

**Table 1:** Kinases with putative phosphorylation consensus sequences in HTT exhibit abnormal associations with HTT in HD.

ProteinAC	Kinase	Description	TOTAL LMs	HTT-IP LMs
P31749	AKT1	AKT serine/threonine kinase 1	DECREASED	GAINED
P49841	GSK3β	glycogen synthase kinase 3 beta	SIG. INCREASED	GAINED
P49840	GSK3α	glycogen synthase kinase 3 alpha	SIG. INCREASED	GAINED
P27361	ERK1/MAPK3	extracellular signal-regulated kinase 1	DECREASED	GAINED
P06493	CDK1	Cyclin-dependent kinase 1	UNCHANGED	SIG. INCREASED
Q00535	CDK5	Cyclin-dependent kinase 5	UNCHANGED	GAINED

Score	Motif	Motif Group	Site	Sequence	Gene Info	Referenced
0.367	GSK3 (motif: S*-X3-pS,+4 S)	Acidophilic serine/threonine kinase group (Acid_ST_kin)	S2657	APSSPPTsPVNSRKH	GSK3β	Schilling et al. [35]
0.405	GSK3 (motif: S*-X3-pS,+4 S)	Acidophilic serine/threonine kinase group (Acid_ST_kin)	S2657	APSSPPTsPVNSRKH	GSK3α	Schilling et al. [35]
0.431	ERK (motif: PX-S*-P)	Proline-dependent serine/threonine kinase group (Pro_ST_kin)	S2076	DSLSPpSPPVSSHPL	ERK1/MAPK3	Schilling et al. [35]
0.541	ERK (motif: PX-S*-P)	Proline-dependent serine/threonine kinase group (Pro_ST_kin)	S2653	APAPSsPPTSPVNSRKH	ERK1/MAPK3	Schilling et al. [35]
0.42	ERK (motif: PX-S*-P)	Proline-dependent serine/threonine kinase group (Pro_ST_kin)	S2657	APSSPPTsPVNSRKH	ERK1/MAPK3	Schilling et al. [35]
0.374	AKT-1 (motif: XXRXRXX-S*-X)	Basophilic serine/threonine kinase group (Baso_ST_kin)	S421	GGRSRSGsIVEL	AKT1	Schilling et al. [35]
0.448	AKT-1 (motif: XXRXRXX-S*-X)	Basophilic serine/threonine kinase group (Baso_ST_kin)	T2066	LDRFRLStMQDSLSP	AKT1	
0.274	CDK1 (motif: X-S*-PXR/K)	Proline-dependent serine/threonine kinase group (Pro_ST_kin)	S1181	LTNPPSLsPIRRKGK	CDK1	Schilling et al. [35]
0.334	CDK5 (motif: X-S*-PK/RXR/K)	Proline-dependent serine/threonine kinase group (Pro_ST_kin)	S1181	LTNPPSLsPIRRKGK	CDK5	Schilling et al. [35]
0.266	CDK1 (motif: X-S*-PXR/K)	Proline-dependent serine/threonine kinase group (Pro_ST_kin)	S1201	EQASVPLsPKKGSEA	CDK1	Schilling et al. [35]
0.372	CDK5 (motif: X-S*-PK/RXR/K)	Proline-dependent serine/threonine kinase group (Pro_ST_kin)	S1201	EQASVPLsPKKGSEA	CDK5	Schilling et al. [35]
0.348	CDK5 (motif: X-S*-PK/RXR/K)	Proline-dependent serine/threonine kinase group (Pro_ST_kin)	T1857	WWAEVQQtPKRHSLS	CDK5	
0.281	CDK1 (motif: X-S*-PXR/K)	Proline-dependent serine/threonine kinase group (Pro_ST_kin)	S2911	VDRVNVHsPHRAMAA	CDK1	
0.327	CDK5 (motif: X-S*-PK/RXR/K)	Proline-dependent serine/threonine kinase group (Pro_ST_kin)	S3124	EVVAAPGsPYHRLLT	CDK5	


List of kinase names and family groups that were present in both HTT-IP and LMs of human iNeurons with which the HTT sequence was previously proposed [35] to contain putative phosphorylation consensus sequences. Note, ERK and GSK3 are both predicted to phosphorylate Ser2657 of HTT, while CDK1 and CDK5 are both predicted to phosphorylate either Ser1181 or Ser1201. A schematic of the HTT protein illustrates the relative locations of these putative phosphorylation sites.

### GSK3β and ERK1 both phosphorylate normal and pathogenic HTT

**Fig. 3:**
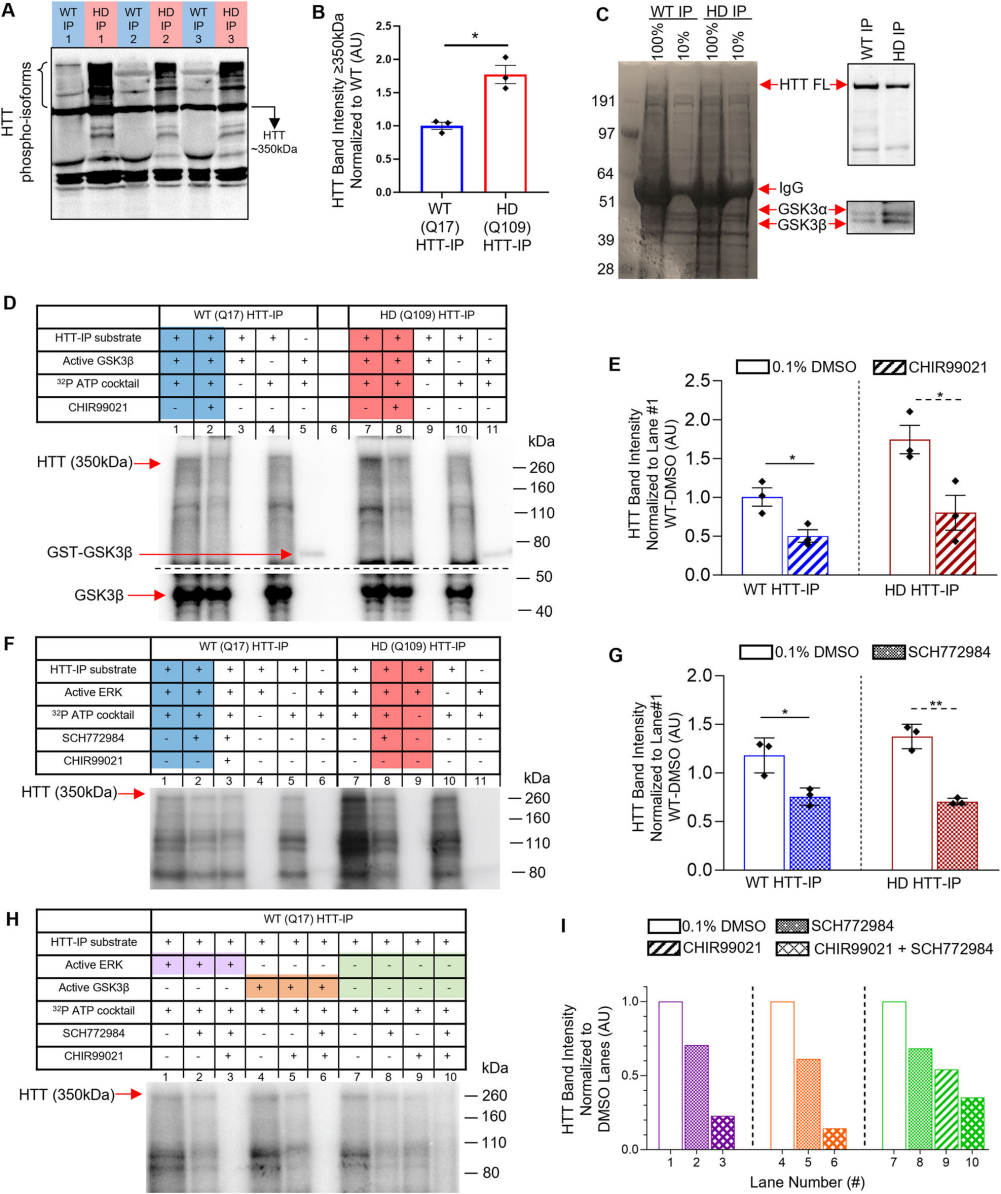
GSK3β and ERK phosphorylate non-pathogenic and pathogenic HTT in vitro. **A** HTT-IPs from WT and HD iNeurons were run on a Zn^2+^-phos-tag™ AAL-107 gel, transferred via western blot, and probed for HTT. Note the increased density of high-molecular weight bands in HD samples indicative of multiple phosphoprotein isoforms that were not present in WT samples, suggesting increased phosphorylation of HTT occurs in HD iNeurons. **B** Quantification of total HTT signal intensity ≥350 kDa, normalized to WT Q17 HTT-IPs. **C** HTT-IPs from WT and HD iNeurons show that increased levels of active GSK3α/β pull down with HTT from HD iNeurons compared to WT. **D** HTT-IPs from WT and HD iNeurons were used as a substrate incubated with GST-GSK3β (~74 kDa) for an in vitro kinase assay in the presence of 1 mCi/100 γ32P-ATP. To account for a baseline level of HTT-phosphorylation by endogenous kinases that IP with HTT, 1 mCi/100 γ32P-ATP and HTT-IP substrate were incubated without GST-GSK3β. The autoradiograph shows a strong band at ~350 kDa, indicating the phosphorylation of HTT by GSK3β that is largely diminished in the presence of CHIR99021, a GSK3β inhibitor. The 350 kDa shows increased intensity with pathogenic HTT from HD iNeurons, indicative of increased GSK3β-mediated phosphorylation of HTT. Note that a strong band is also observed at ∼47 kDa supporting previous findings that GSK3β can auto-phosphorylate itself. *n* = 3. **E** Quantification of the band intensity at 350 kDa from WT or HD HTT-IPs in the presence/absence of CHIR99021. **F** HTT-IPs from WT and HD iNeurons were used as a substrate incubated with GST-ERK1 (~72 kDa) for an in vitro kinase assay in the presence of 1 mCi/100 γ32P-ATP. To account for a baseline level of HTT-phosphorylation by endogenous kinases that IP with HTT, 1 mCi/100 γ32P-ATP and HTT-IP substrate were incubated without GST-ERK1. The autoradiograph shows a strong band at ∼350 kDa, indicating the phosphorylation of HTT by ERK1 that is largely diminished in the presence of SCH772984, an ERK1/2 inhibitor. The 350 kDa shows increased intensity with pathogenic HTT from HD iNeurons, indicative of increased ERK1-mediated phosphorylation of HTT. *n* = 3. **G** Quantification of the band intensity at 350 kDa from WT or HD HTT-IPs in the presence/absence of SCH772984. **H** HTT-IPs from WT iNeurons were used as a substrate incubated with GST-GSK3β or GST-ERK1 for an in vitro kinase assay in the presence of 1 mCi/100 γ32P-ATP. To account for a baseline level of HTT-phosphorylation by endogenous kinases that IP with HTT, 1 mCi/100 γ32P-ATP and HTT-IP substrate were incubated without GST-GSK3β or GST-ERK1. The autoradiograph shows a strong band at ∼350 kDa that is largely diminished in the presence of CHIR99021 or SCH772984. **I** Quantification of the band intensity at 350 kDa from WT HTT-IPs normalized to DMSO-only lanes (lane#1, #4, or #7). Statistical analysis was conducted using the two-sample two-sided Student’s *t* test comparing signal/noise intensity between bands in WT and HD conditions normalized to WT. Data represented as mean ± SEM. ns = *p* > 0.05, *p < 0.05, **p < 0.005.

HD progression is often characterized by cytoplasmic/nuclear inclusions, correlated with loss of striatal neurons, and clinical decline [66, 67]. Synaptic plasticity/transmission deficiencies [68–71] and defective axonal transport [30, 72, 73] have been proposed to occur early in disease progression, with transport defects arising prior to behavioral defects in mice expressing full-length HTT with expanded polyQ repeats [74]. Therefore, since pathogenic HTT has been shown to have elevated phosphorylation levels compared to non-pathogenic HTT [26, 53, 75], changes in the phosphorylation state of HTT could contribute to neuronal dysfunction observed in HD. Indeed, significantly increased levels of phospho-isoforms of HTT were seen in HD iNeurons compared to normal/WT (Fig. 3A, B). Using replicate HTT IPs from the LMs from normal/WT or HD iNeurons that were subjected to LC-MS (Fig. 3C), we conducted in vitro kinase phosphorylation assays to test the prediction that GSK3β and/or ERK1 phosphorylate HTT. A ~350 kDa band was visible after incubating the HTT-IP with recombinant GST-tagged GSK3β and ^32^P ATP, suggesting that both normal and pathogenic HTT is phosphorylated by GSK3β (Fig. 3D-lanes 1,7). The addition of a GSK3β specific inhibitor, CHIR99021 [76], which we previously used in [77], significantly reduced the levels of HTT phosphorylation (Fig. 3D, E-lanes 2,8). However, complete inhibition was not seen as likely due to the presence of endogenous GSK3β and other kinases in the HTT-IP (Figs. 3C and 2E).

A ~ 350 kDa band was also visible after incubating HTT IP from normal/WT or HD iNeurons with recombinant GST-tagged ERK1 and ^32^P ATP, suggesting that both normal and pathogenic HTT is phosphorylated by ERK1 (Fig. 3F-lanes 1,8). Adding an ERK-specific inhibitor, SCH772984 [78], significantly reduced the levels of HTT phosphorylation (Fig. 3F, G-lanes 2, 8), but not completely, perhaps due to the presence of endogenous ERK1 and other kinases in the HTT-IP (Fig. 2E). To access the coordination of both GSK3β and ERK1 phosphorylation events on HTT, we combined both CHIR99021 and SCH772984 inhibitors in the absence of GST-tagged GSK3β or GST-tagged ERK1 and failed to observe complete elimination of HTT phosphorylation (Fig. 3H, I-lane 10), suggesting that other kinases, perhaps AKT1, present in the HTT-IP (Fig. 2E and Tables 1, S1 and S2) phosphorylates HTT. The Coomassie-stained gels show equal protein levels (Fig. S5A–C), suggesting that the observed difference in phosphorylation is likely not due to changes in the number of total proteins loaded. Taken together, our observations identify that both GSK3β and ERK1 can phosphorylate normal and pathogenic HTT. However, the biological relevance of GSK3β- or ERK1-mediated phosphorylation of HTT and how these phosphorylation events contribute to HD is unknown.

### GSK3β-dependent events on HTT contribute to axonal transport and synaptic morphological defect, and neuronal cell death seen in HD

**Fig. 4:**
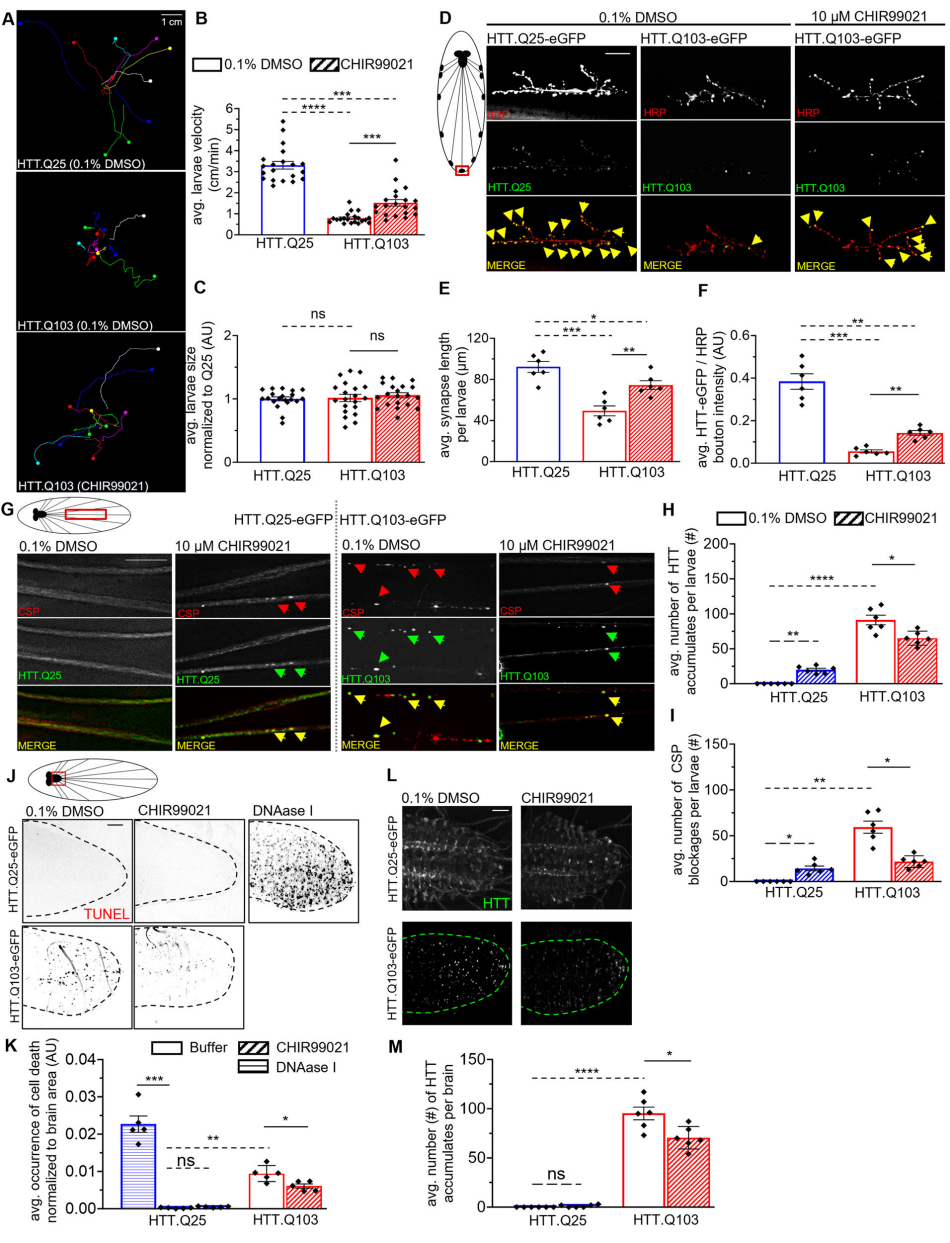
GSK3 inhibition mitigates larval locomotion defects, axonal transport blockages, abnormal synaptic morphology, and elevated neuronal cell death elicited by pathogenic HTT. **A** HTT.Q103-eGFP expressing larva that was fed fly food laced with either 0.1% DMSO or 10 µM CHIR99021 for 24 h were subjected to larval crawling assays for 1.5 mins at 25 °C, 60% humidity and compared to DMSO-treated HTT.Q25-eGFP larvae. *n* = 20. Bar = 1 cm. **B** Quantification of the avg. larval crawling velocity (cm/min) comparing CHIR99021-feeding to DMSO-treatment of HTT.Q103-eGFP expressing larvae and DMSO-treated HTT.Q25-eGFP expressing larvae. **C** Quantification of avg. larval size (area; cm^2^) normalized to HTT.Q25 expressing larvae (AU) comparing CHIR99021-feeding to DMSO-treatment of HTT.Q103-eGFP expressing larvae and DMSO-treated HTT.Q25-eGFP expressing larvae. **D** Schematic of *Drosophila* larval nervous system showing brain, segmental nerves, and neuromuscular junctions (NMJs). Red box = imaged area. Representative images of NMJs from muscle 6/7 segment A4–5 of HTT.Q25-eGFP or HTT.Q103-eGFP expressing larva that were fed fly food laced with either 0.1% DMSO or 10 µM CHIR99021 for 24 h prior to dissection and staining with HRP-TxRED. Top panels = HRP-TxRED staining. Bottom Panel = HTT-eGFP fluorescence. *n* = 6 larvae. Bar = 15μm. Note an even distribution of colocalization (yellow arrows) between HTT.Q25 (green) at boutons (red) is observed, which is lost in the HTT.Q103 expressing larva. **E** Quantification of the avg. synapse length (μm) per larvae comparing CHIR99021-feeding to DMSO-treatment of HTT.Q25-eGFP or HTT.Q103-eGFP expressing larvae. **F** Quantification of the avg. HTT-eGFP (green) signal intensity at boutons normalized to HRP (red) signal intensity (AU) per larvae comparing CHIR99021-feeding to DMSO-treatment of HTT.Q25-eGFP or HTT.Q103-eGFP expressing larvae. **G** Schematic of *Drosophila* larval nervous system showing brain and segmental nerves. Red box = imaged area. Representative larval segmental nerves stained with the synaptic vesicle marker Cysteine String Protein (CSP) from HTT.Q25-eGFP (non-pathogenic) or HTT.Q103-eGFP (pathogenic) expressing larva that were fed fly food laced with either 0.1% DMSO or 10 µM CHIR99021 for 24 h prior to dissections. *n* = 6 larvae. Bar = 10 µm. Note while DMSO-treated larvae expressing HTT.Q25-eGFP show smooth staining in larval nerves, HTT.Q103-eGFP expressing larva show axonal blockages (white arrows) that stain with CSP and contain HTT. **H**, **I** Quantification of the average number (#) of axonal HTT **H** accumulations or **I** CSP blockages within larval segmental nerves in CHIR99021-feed compared to DMSO-fed HTT.Q25-eGFP or HTT.Q103-eGFP expressing larvae. **J** Representative larval brains evaluated for cell death using TUNEL-labelling of HTT.Q25-eGFP or HTT.Q103-eGFP expressing larva that were fed fly food laced with either 0.1% DMSO or 10 µM CHIR99021 for 24 h. Red box in schematic = imaged are. *n* = 5 larvae. Bar = 10 µm. Larval brains expressing HTT.Q103-eGFP show TUNEL-positive cells, in contrast to HTT.Q25-eGFP expressing larvae. However, HTT.Q25 expressing larvae show robust TUNEL-positive cells in the context of 1 µg/µL DNAase I (positive-control). **K** Quantification of the average number (#) of TUNEL-positive cells normalized to imaged brain area comparing CHIR99021-feeding to DMSO-treatment of HTT.Q25-eGFP or HTT.Q103-eGFP expressing larvae. **L** Representative larval brains were evaluated for aggregations of HTT in HTT.Q25-eGFP or HTT.Q103-eGFP expressing larva that were fed fly food laced with either 0.1% DMSO or 10 µM CHIR99021 for 24 h. *n* = 5 larvae. Bar = 10 µm. **M** Quantification of the average number (#) of HTT accumulates/aggregates per larval brain comparing CHIR99021-feeding to DMSO-treatment of HTT.Q25-eGFP or HTT.Q103-eGFP expressing larvae. Statistical significance was determined using the two-sample two-sided Student’s *t* test. Data represented as mean ± SEM. ns = *p* > 0.05, *p < 0.01, **p < 0.001, ***p < 0.0001, ****p < 0.00001.

Given that GSK3β phosphorylates both normal and mutant HTT (Fig. 3), and previous MS-based findings identified a putative GSK3β phosphorylation site at Ser2657 (Table 1, [35]), we next tested the hypothesis that active GSK3β contributes to HD pathogenesis. To do so, we employed a humanized *Drosophila* HD model to assess GSK3β-dependent effects in a whole organism. Similar to differentiated neurons from HD patient-derived iPSCs, larval segmental neurons from larvae expressing pathogenic HTT contained axonal blockages that stained with HTT and synaptic proteins [33], show disrupted transport of RAB proteins [32, 34], accumulation of HTT/polyQ aggregates and cell death [30, 79, 80] indicating that both human HD iNeurons and *Drosophila* neurons expressing pathogenic HTT show similar dysfunctions. In *Drosophila,* a specialized neuronal network known as the central pattern generator coordinates the peristaltic movement of larval muscles, resulting in wave-like contractions along the posterior-to-anterior axis of the larvae to facilitate larval crawling [81–83]. We previously showed that larvae expressing pathogenic human HTT had severe locomotor defects together with axonal blockages within their axons and synaptic morphological defects at their neuromuscular junctions (NMJs) [33, 34, 79]. To test how active GSK3β influences these phenotypes, we first generated larvae expressing either non-pathogenic normal HTT (HTTex1.Q25-eGFP) or disease-causing, pathogenic HTT (HTTex1.Q103-eGFP) using the pan-neuronal driver APPL-GAL4 and inhibited GSK3β by feeding larvae on food laced with either 10 µM CHIR99021, a potent inhibitor of GSK3β and an inducer of the Wnt/beta-catenin pathway [76], or 0.1% DMSO for 24 h prior to the behavioral studies. CHIR99021 was previously characterized in *Drosophila* S2 (Kc167) cells showing activated Wnt signaling at 12.5 µM and 25 µM CHIR99021 after 24 h, but not at 6.3 µM [84]. We previously showed that 10 µM CHIR99021 but not 5 µM was sufficient to produce an axonal blockage phenotype similar to larvae expressing dominant negative *shaggy* (*sgg*), the *Drosophila* homolog of *GSK3β* [77, 85]. Higher concentrations of CHIR99021 led to embryonic lethality, comparable to homozygous loss of function *sgg* mutants [86, 87]. Consistent with our previous observations [33] larvae expressing pathogenic HTT (Q103) showed significant larval locomotor defects compared to non-pathogenic normal HTT (Q25; Fig. 4A, B). Surprisingly, larvae cultured on food laced with the GSK3β inhibitor (CHIR99021) showed significantly attenuated locomotion defects (Fig. 4A, B). However, larval growth was not affected by pathogenic HTT expression or feeding food laced with CHIR99021 (Fig. 4C).

To examine how active GSK3β contributes to pathogenic HTT-mediated axonal transport defects and synaptic growth defects, we evaluated type-1 synaptic boutons in NMJs between muscle 6/7 at larval abdominal segments A4-A5 as we had previously done [33, 34, 79, 88]. We found that CHIR99021 feeding alleviated the synaptic growth defects observed in larval NMJs expressing pathogenic HTT (Q103; Fig. 4D, E). Notably, concomitant increases in the levels of HTT at synapses were seen in these larvae (Fig. 4D, F) compared to DMSO-fed larvae, suggesting that the movement of HTT to NMJs is likely regulated by active GSK3β. Since larval locomotor defects correlate with the number of axonal blockages [88] and axonal transport defects caused by loss of motor proteins or excess linker/receptor proteins result in synaptic defects [79], we next tested the proposal that GSK3β plays a role in pathogenic HTT-mediated axonal transport defects. As expected, DMSO-fed larvae expressing pathogenic HTT (Q103) showed axonal blockages containing both CSP (cysteine string protein) and HTT, but CHIR99021-fed larvae showed significantly decreased numbers of CSP and HTT-containing axonal blockages (Fig. 4G–I). Unsurprisingly CHIR99021-fed larvae expressing normal/non-pathogenic HTT (Q25) also caused axonal blockages that contained both CSP and HTT. Although the extent of these blockages was not as comparable to those seen with pathogenic HTT expression, the amounts of blockages were significant compared to DMSO-fed controls. These observations are similar to what was observed for GSK3β loss of function mutants [86] and confirm GSK3β’s role in the axonal transport of synaptic vesicles [42, 87]. Therefore, while inhibition of GSK3β can cause axonal transport defects by decreasing the number of motors associated with synaptic membranes [42], these findings implicate active GSK3β in escalating pathogenic HTT-mediated axonal transport defects, either by directly phosphorylating HTT (Fig. 4D, E) and/or by regulating the recruitment of motor proteins onto CSP- and/or HTT-containing membranes [77, 85]. Indeed, this proposal is validated via larvae expressing excess *sgg* with pathogenic HTT, which still showed brain accumulations, axonal blockages, synaptic morphology defects, and decreased levels of HTT at NMJs (Fig. S6)

Previously, we showed that the extent of axonal blockages correlated with the extent of HTT aggregates and cell death [30, 80]. However, the mechanism by which pathogenic HTT initiates cell death remains obscure. To test how active GSK3β affects pathogenic HTT-mediated neuronal cell death, we examined larval brains expressing pathogenic HTT (Q103) and normal HTT (Q25) in the context of the GSK3β inhibitor CHIR99021. While expression of pathogenic HTT (Q103) caused significant amounts of cell death in larval brains, CHIR99021-fed larval brains showed significant reductions in cell death (Fig. 4J, K). Significantly decreased levels of HTT accumulations within larval brains (Fig. 4L, M) were also seen, perhaps due to stimulating lysosomal degradation of HTT aggregates as reported previously in mice brains [58]. In contrast, larvae expressing excess *sgg* with pathogenic HTT did not change the levels of HTT accumulations within larval brains (Fig. S6A). Therefore, excess active GSK3β likely causes neuronal cell death through multiple pathways, including those that are triggered by phosphorylation of HTT and/or a cascade of events activated by pathogenic HTT-mediated transport defects.

### ERK-dependent events on HTT are likely neuroprotective against pathogenic HTT-mediated neuronal cell death

**Fig. 5:**
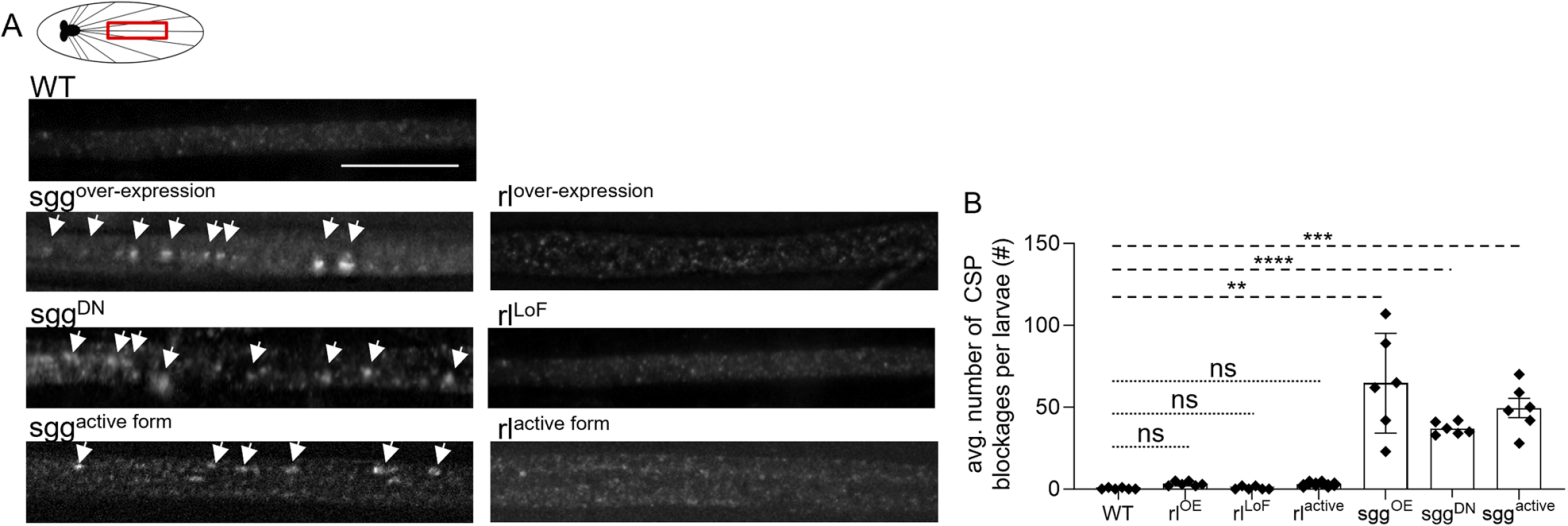
ERK does not play a major role in axonal transport regulation, unlike GSK3β. **A** Schematic of *Drosophila* larval nervous system showing brain and segmental nerves. Red box = imaged area. Representative larval segmental nerves from WT, *sgg*^OE^, *sgg*^DN^, *sgg*^active^, *rl*^OE^,*rl*^LoF^, or *rl*^active^ larva stained with the synaptic vesicle marker CSP. *n* = 6 larvae. Bar = 10 µm. OE=overexpression, LOF= loss of function, DN= dominant negative, Active=constitutively active. **B** Quantification of the average number (#) of axonal CSP blockages per larvae within segmental nerves. Statistical significance was determined using the two-sample two-sided Student’s *t* test. Data represented as mean ± sem. ns = *p* > 0.01, *p < 0.01, **p < 0.001, ***p < 0.0001, ****p < 0.00001.

A MS-based finding proposed three putative ERK phosphorylation sites on HTT (Table1, [35]). However, the functional significance of ERK-dependent events on HTT remains ambiguous. Consistent with our observations in HD iNeurons, cholinergic and somatostatinergic interneurons of the striatum in R6/2 mice showed higher levels of ERK compared to controls [89]. Phosphorylated ERK levels also increased with age in these mice [89]. However, work in a striatal cell line derived from a transgenic homozygous knock-in mouse with 111CAG repeats (STHdhQ111/Q111) showed no change [90]. In contrast to GSK3β [58], ERK-mediated signaling was suggested to be neuroprotective in the context of pathogenic HTT expression [55, 56, 91, 92], but the mechanisms are unknown. Unlike GSK3β [77, 85, 86, 93], little is currently known about the role of ERK within the axonal transport pathway despite ERK being shown to phosphorylate dynein intermediate chain (DIC) at Ser80 [94]. Therefore, to first test whether ERK functions during axonal transport, we assessed larvae carrying a hypomorphic allele for *rolled (rl)*, the *Drosophila* homolog of ERK, and larvae expressing excess wild-type *rl* or active *rl* [95]. Previous work showed that *rl* mutant larvae exhibit aberrant nerve innervation patterns in abdominal body-wall muscles and premature defasciculation of axonal bundles [96]. Furthermore, *rl* mutant flies have a rough eye phenotype due to missing photoreceptor cells [97]. Surprisingly, neither condition (hypomorphic nor excess) showed axonal transport defects (Fig. 5A, B), which is in contrast to larvae expressing excess wildtype, excess active, or excess dominant negative *sgg*, which showed significant axonal blockages (Fig. 5A, B; [85]). Therefore, unlike GSK3β, ERK likely has no major role during axonal transport.

**Fig. 6:**
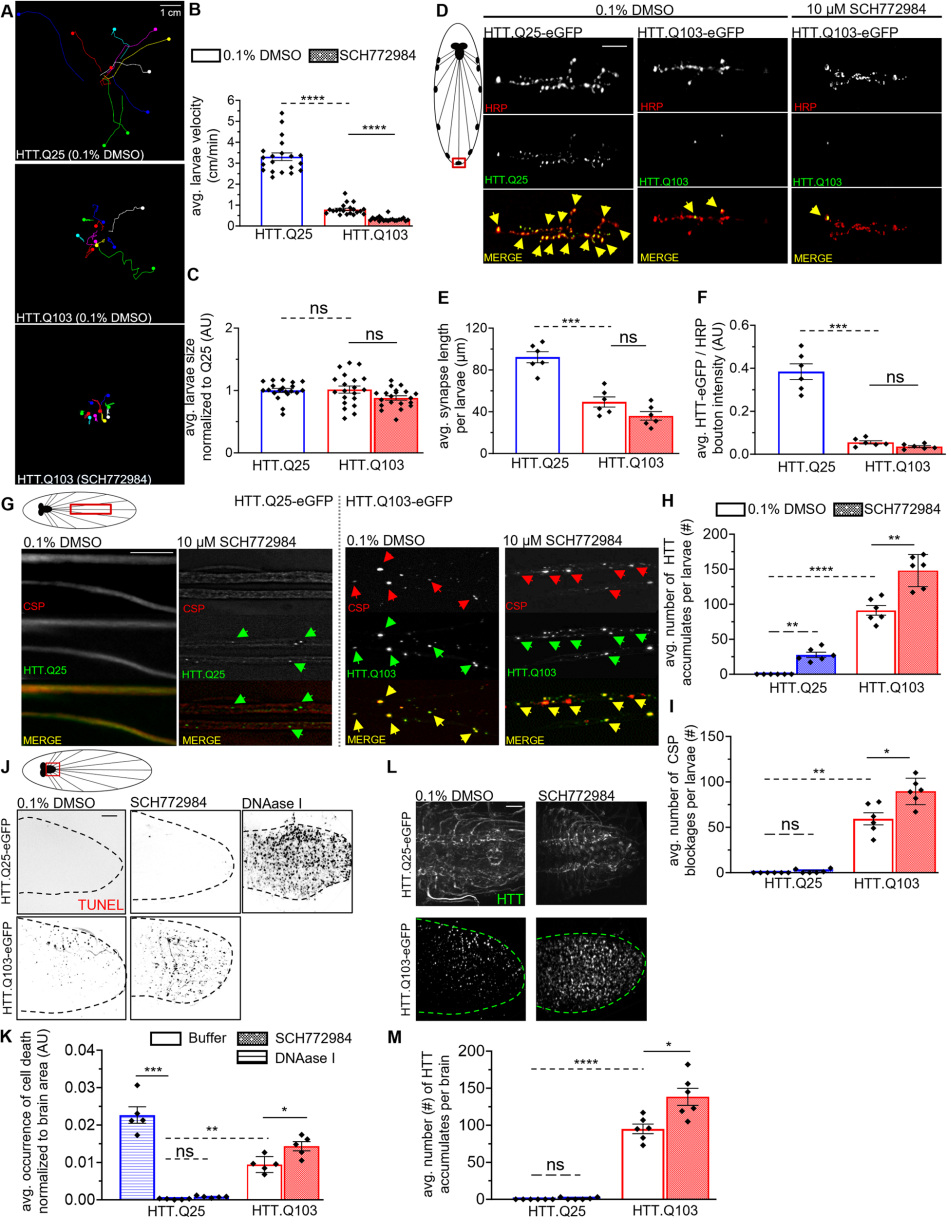
ERK inhibition causes accumulation of non-pathogenic HTT in larval nerves and exacerbates pathogenic HTT-induced neuronal cell death. **A** HTT.Q103-eGFP expressing larva that were fed fly food laced with either 0.1% DMSO or 10 µM SCH772984 for 24 h were subjected to larval crawling assays for 1.5 mins at 25 °C, 60% humidity and compared to DMSO-treated HTT.Q25-eGFP larvae. *n* = 20. Bar = 1 cm. **B** Quantification of the avg. larval crawling velocity (cm/min) comparing SCH772984-feeding to DMSO-treatment of HTT.Q103-eGFP expressing larvae and DMSO-treated HTT.Q25-eGFP expressing larvae. **C** Quantification of avg. larval size (area; cm^2^) normalized to HTT.Q25 expressing larvae (AU) comparing SCH772984-feed to DMSO-fed HTT.Q103-eGFP expressing larvae and DMSO-fed HTT.Q25-eGFP expressing larvae. **D** Schematic of *Drosophila* larval nervous system showing brain, segmental nerves, and neuromuscular junctions (NMJs). Red box = imaged area. Representative images of NMJs from muscle 6/7 segment A4–5 of HTT.Q25-eGFP or HTT.Q103-eGFP expressing larva that were fed fly food laced with either 0.1% DMSO or 10 µM SCH772984 for 24 h prior to dissection and stained with HRP-TxRED. Top panels = HRP-TxRED staining. Bottom Panel = HTT-eGFP fluorescence. *n* = 6 larvae. Bar = 15μm. Note an even distribution of colocalization (yellow arrows) between HTT.Q25 (green) and HRP (red) at boutons is observed, which is lost in HTT.Q103 expressing larva. **E** Quantification of the avg. synapse length (μm) per larvae comparing SCH772984-fed to DMSO-fed HTT.Q25-eGFP or HTT.Q103-eGFP expressing larvae. **F** Quantification of the avg. HTT-eGFP (green) signal intensity at boutons normalized to HRP (red) signal intensity (AU) per larvae comparing SCH772984-fed to DMSO-fed HTT.Q25-eGFP or HTT.Q103-eGFP expressing larvae. **G** Schematic of *Drosophila* larval nervous system showing brain and segmental nerves. Red box = imaged area. Representative larval segmental nerves stained with the synaptic vesicle marker CSP from HTT.Q25-eGFP (non-pathogenic) or HTT.Q103-eGFP (pathogenic) expressing larvae that were fed fly food laced with either 0.1% DMSO or 10 µM SCH772984 for 24 h prior to dissections. *n* = 6 larvae. Bar = 10 µm. Note while DMSO-fed larvae expressing HTT.Q25-eGFP show smooth staining in larval nerves, HTT.Q103-eGFP expressing larvae show axonal blockages (white arrows) that stain with CSP and contain HTT. **H**, **I** Quantification of the average number (#) of axonal **H** HTT accumulations or **I** CSP blockages within larval segmental nerves comparing SCH772984-fed to DMSO-fed HTT.Q25-eGFP or HTT.Q103-eGFP expressing larvae. **J** Red box = imaged are. Representative larval brains were evaluated for cell death using TUNEL-labelling of HTT.Q25-eGFP or HTT.Q103-eGFP expressing larvae that were fed fly food laced with either 0.1% DMSO or 10 µM SCH772984 for 24 h. *n* = 5 larvae. Bar = 10 µm. Larval brains expressing HTT.Q103-eGFP show TUNEL-positive cells, in contrast to HTT.Q25-eGFP expressing larvae. However, HTT.Q25 expressing larvae show robust TUNEL-positive cells in the context of 1 µg/µL DNAase I (positive-control). **K** Quantification of the average number (#) of TUNEL-positive cells normalized to the imaged brain area comparing SCH772984-fed to DMSO-fed HTT.Q25-eGFP or HTT.Q103-eGFP expressing larvae. **L** Representative larval brains evaluated for aggregations of HTT in HTT.Q25-eGFP or HTT.Q103-eGFP expressing larva that were fed fly food laced with either 0.1% DMSO or 10 µM SCH772984 for 24 h. *n* = 5 larvae. Bar = 10 µm. **M** Quantification of the average number (#) of HTT accumulates/aggregates per larval brain comparing SCH772984-fed to DMSO-fed HTT.Q25-eGFP or HTT.Q103-eGFP expressing larvae. Statistical significance was determined using the two-sample two-sided Student’s *t* test. Data represented as mean ± sem. ns = *p* > 0.01, *p < 0.01, **p < 0.001, ***p < 0.0001, ****p < 0.00001.

To test the prediction that ERK activity can specifically influence the movement of HTT within axons, we examined larvae expressing non-pathogenic (Q25) and pathogenic (Q103) HTT fed on food laced with 10 µM SCH772984, which inhibits ERK1/2 [78] and on 0.1% DMSO-laced food. We found that feeding 10 µM SCH772984 was not toxic to WT flies in contrast to feedings at 25 µM. In contrast to GSK3β inhibition, inhibition of ERK enhanced pathogenic HTT-mediated larval locomotor deficits (Fig. 6A–C), and increased axonal blockages containing HTT and CSP (Fig. 4G–I). However, no defects were observed at NMJs or synapses (Fig. 4D–F). Inhibition of ERK significantly increased neuronal cell death (Fig. 4J, K), concomitant with significant increases in HTT accumulations within pathogenic HTT-expressing larval brains (Fig. 4L, M). Strikingly, and in contrast to GSK3β inhibition, ERK inhibition did not induce CSP-containing axonal blockages but caused a significant number of HTT-containing blockages in larvae expressing non-pathogenic (Q25) HTT. Taken together, these observations suggest that while overall ERK likely exerts a neuroprotective role, ERK’s effect on axonal transport is restricted to HTT, in contrast to GSK3β, which has a broader role during axonal transport.

**Fig. 7:**
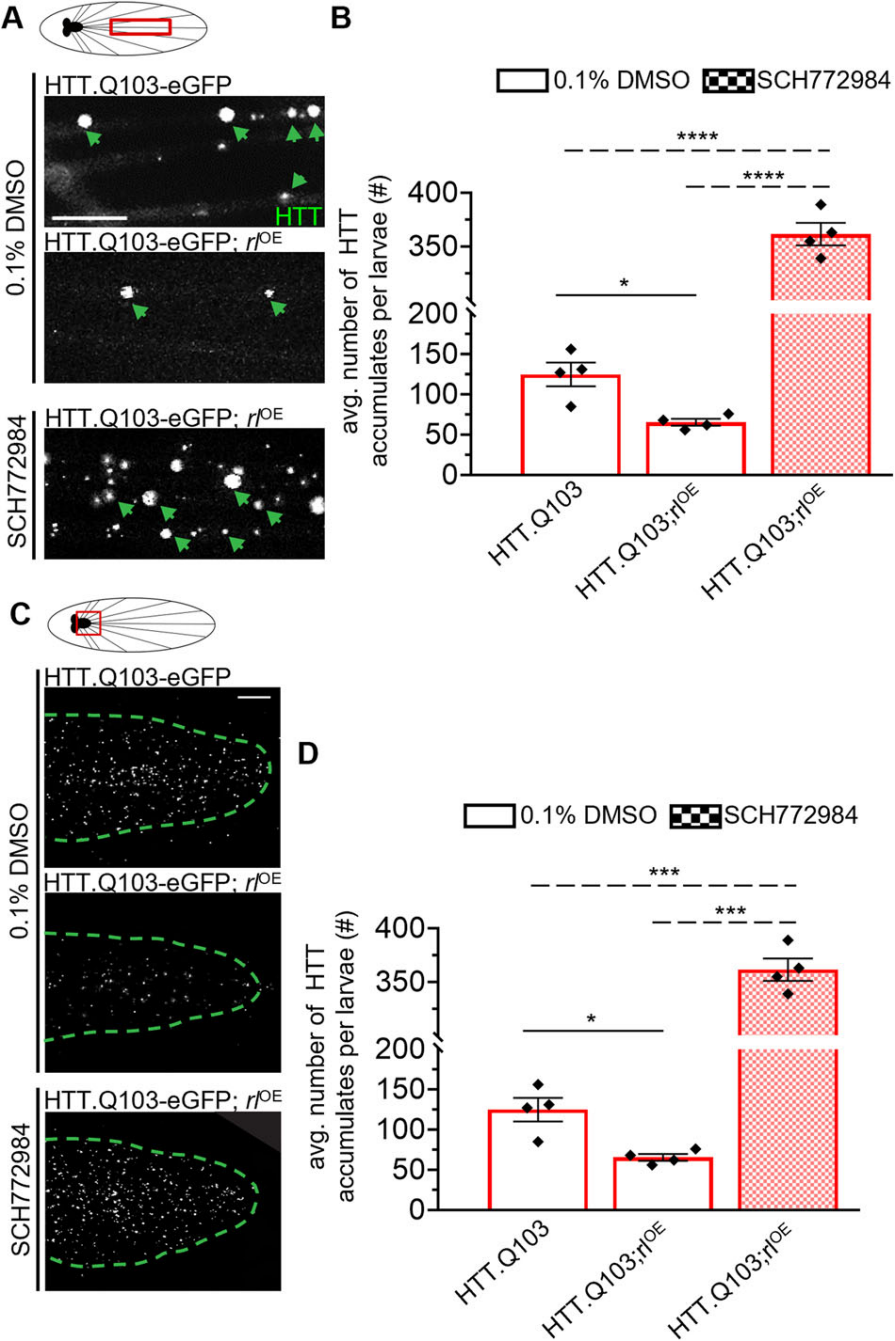
Excess ERK rescues accumulations of pathogenic HTT in larval nerves and brains. **A** Schematic of *Drosophila* larval nervous system showing brain and segmental nerves. Red box = imaged area. Representative larval segmental nerves from HTT.Q103-eGFP or HTT.Q103-eGFP; UAS-rl^OE^ expressing larva that were fed fly food laced with either 0.1% DMSO or 10 µM SCH772984 for 24 h prior to dissections. *n* = 4 larvae. Bar = 10 µm. Note, HTT.Q103-eGFP expressing larva show axonal accumulations (arrows). **B** Quantification of the average number (#) of axonal HTT accumulations within larval segmental nerves comparing SCH772984-feeding to DMSO-treatment of HTT.Q103-eGFP or HTT.Q103-eGFP; UAS-rl^OE^ expressing larvae. **C** Schematic of *Drosophila* larval nervous system showing brain and segmental nerves. Red box = imaged area. Representative larval brain from HTT.Q103-eGFP or HTT.Q103-eGFP; UAS-rl^OE^ expressing larva that were fed fly food laced with either 0.1% DMSO or 10 µM SCH772984 for 24 h prior to dissections. *n* = 4 larvae. Bar = 10 µm. **D** Quantification of the average number (#) of HTT accumulations within larval brains comparing SCH772984-fed to DMSO-fed HTT.Q103-eGFP or HTT.Q103-eGFP; UAS-rl^OE^ expressing larvae. Statistical significance was determined using the two-sample two-sided Student’s *t* test. Data represented as mean ± sem. ns = *p* > 0.01, *p < 0.01, **p < 0.001, ***p < 0.0001. ****p < 0.00001.

**Fig. 8:**
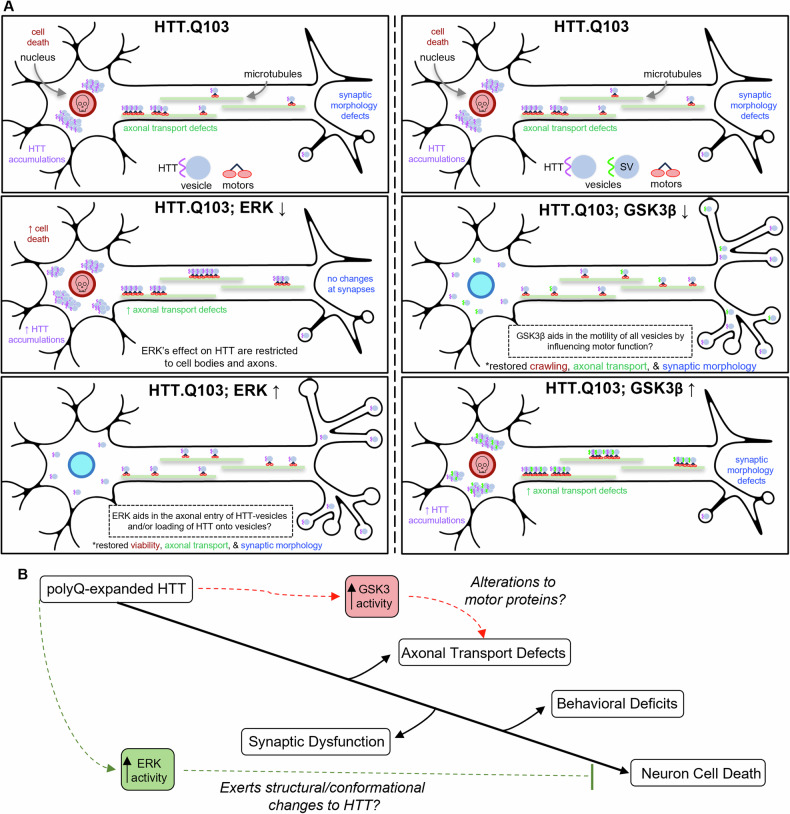
Schematic model for how ERK and GSK3β levels modulates HD pathogenesis. **A** Changing levels of ERK (left) or GSK3β (right) directly affects pathogenic HTT-mediated neuropathology. Expression of pathogenic HTT causes, HTT accumulations and cell death at cell bodies, HTT blockages that disrupt axonal transport and synaptic defects. Expression of pathogenic HTT with either decreased ERK or excess GSK3β enhances these phenotypes. However, expression of pathogenic HTT with either decreased GSK3β or adding excess ERK alleviates these phenotypes and restores axonal transport, synaptic morphology, and/or neuronal viability. **B** In HD, pathogenic HTT causes axonal transport defects and synaptic dysfunction that gives rise to locomotor deficits and neuronal cell death. While elevated GSK3β activity may exacerbate pathogenic HTT-mediated axonal transport defects via alternations to motor protein function and synaptic dysfunction via decreases in axonal transport of essential cargo, elevated ERK activity is likely neuroprotective and curtails neuronal cell death caused by aberrant structural and conformational changes to HTT.

To validate our prediction that ERK has a protective role in the context of pathogenic HTT-mediated neuronal dysfunction, we added wild-type ERK with pathogenic HTT. Co-expression of *rl* with HTT-Q103 rescued pathogenic HTT axonal blockages within larval nerves (Fig. 7A, B) and HTT accumulations within larval brains (Fig. 7C, D). However, feeding these larvae food laced with the ERK inhibitor SCH772984 reverted these phenotypes (Fig. 7A–D), indicating that changing the level of ERK can directly contribute to HTT-mediated pathogenic phenotypes (Fig. 8A). Taken together, our observations support a role for ERK during HD neuronal dysfunction, and propose a mechanism in which ERK activity is likely neuroprotective against pathogenic HTT-mediated neuronal cell death via the depletion of HTT accumulations and rescue of cell death.

## Discussion

Previous studies have unmasked HTT’s role in many cellular processes such as intracellular trafficking, however how HTT affects membrane-associated functions likely vital for both normal neuronal physiology and HD pathogenesis have been overlooked. Our study bridges this gap in knowledge, revealing novel membrane-specific proteomic alterations induced by pathogenic HTT. Specifically, we found significant remodeling of the HTT-associated membrane proteome in HD patient-derived iNeurons (Fig. 1), with notable shifts in key kinases such as mTOR, AKT1, ERK1/2, CDK1/5, and GSK3α/β (Fig. 2). We further discovered that GSK3β and ERK kinases are capable of phosphorylating HTT under both normal and disease conditions (Fig. 3), a phenomenon previously unknown. Further, GSK3β and ERK likely have opposing roles during HD pathogenesis, with GSK3β inhibition rescuing pathogenic HTT-mediated axonal transport defects, synaptic dysfunction, and neuronal cell death (Fig. 4), and ERK inhibition enhancing these phenotypes (Fig. 6) and excess ERK rescuing HTT-containing accumulations caused by pathogenic HTT (Fig. 7). Moreover, unlike GSK3β, ERK had little-to-no effect on other synaptic vesicle transport (Fig. 5). Together our findings unveil a previously unknown role for GSK3β and ERK1 (Fig. 8A) as phospho-regulators of HTT at membranes (Fig. 8B), shedding new light on the pathogenic mechanisms underlying HD and offering a promising highly druggable pathway for therapeutic intervention that can be inhibited by already available small molecules.

### GSK3β and ERK are likely phospho-regulators of HTT

HTT exhibits a remarkable capacity for intramolecular interactions, giving rise to an array of three-dimensional conformations [98]. This versatility is evidenced by the observation of over one hundred structurally distinct conformations [99], which likely aid in HTT’s ability to associate with hundreds of different proteins [9, 10, 12]. Post-translational modifications, such as phosphorylation, could represent potential mechanisms to modulate these intramolecular interactions within HTT, thereby influencing its associations with proteins and membranes, leading to diverse cellular localizations. Indeed, normal HTT is phosphorylated, but pathogenic HTT exhibits hyperphosphorylation ([26, 50, 75], Fig. 3A, B). While phosphorylation at Ser13/Ser16 by IKK increased HTT turnover [100], phosphorylation at either Ser13 or Ser16 also promoted nuclear localization [101, 102] and inhibited membrane binding [103]. However, disruption of the Ser13 and Ser16 sites in BACHD mice did not change HTT’s subcellular location, highlighting those other regions of HTT can play a role in its subcellular localization. Further, membrane interactions and intramolecular interactions within HTT have been proposed to be regulated by AKT (S421; [37]) or CDK5 (S434, S1181, S1201; [59]). While the mechanisms governing how these phosphorylation events impact HTT’s interaction with membranes are ambiguous, the functional significance of other potential HTT phosphorylation sites identified through MS remains unexplored [35, 36].

Here, we identified that GSK3β and ERK1 are both associated with and are capable of phosphorylating normal and pathogenic HTT (Fig. 3). Interestingly, while ERK has three putative phosphorylation sites (Ser2076, Ser2653, and Ser2657), GSK3β has only a sole putative phosphorylation site (Ser2657) at the C-terminus of HTT that overlaps with one of the three putative ERK1 sites [35]. This suggests that there is likely crosstalk between these two kinases, and perhaps a regulatory mechanism exists. While a handful of studies have investigated GSK3β [54, 104–106] and ERK [107] in the context of HD, little is known about functional interactions between these kinases and HTT. Our discovery that GSK3β and ERK1 phosphorylates HTT at membranes proposes a novel mechanism for HD pathogenesis whereby these phosphorylation events become dysfunctional in HD, contributing to neuronal degeneration and cell death. One possible mechanism involves dysregulation of the regulatory interplay between pathways involving GSK3β and ERK1. Indeed, expression of excess or constitutively active GSK3β induces apoptosis in rat cortical neurons, which can be inhibited by expression of constitutively active ERK [65]. However, unlike AKT1, which directly inhibits GSK3β via phosphorylation at Ser9 [64], suppression of GSK3β-mediated apoptosis in rat cortical neurons by ERK was independent of GSK3β phosphorylation at Ser9 (inactivation) or at Tyr216 (activation), suggesting a novel regulatory mechanism between these two kinases. We propose that the overlapping phosphorylation site at Ser2657 can play a role in regulating GSK3β and ERK-mediated functions. Indeed, inhibition of GSK3β (Fig. 4) or excess ERK (Fig. 7) rescued HD phenotypes and decreased axonal transport defects and HTT aggregation, in contrast to inhibition of ERK (Fig. 6) or excess GSK3β (Fig. S6). Previous work also supports this proposal since neuronal dysfunction in HD was ameliorated by either decreased GSK3β-mediated signaling [58] or increased ERK-mediated signaling [55].

### ERK specifically regulates HTT functions, while GSK3β coordinates universal axonal transport

Axonal transport is a tightly regulated cellular process essential for neuron survival. Both HTT and GSK3β have independently emerged as coordinators of axonal transport in the context of motor proteins such as kinesin-1. While HTT acts as a scaffold protein and can interact with kinesin light chain (KLC) via huntingtin-associated protein 1 (HAP1; [108]), AKT1 phosphorylation of HTT at serine 421 is thought to enhance the recruitment of kinesin-1 to vesicles and microtubules, thereby promoting anterograde transport [38]. In contrast, GSK3β phosphorylation can promote motor-membrane associations [85], and GSK3β phosphorylation of kinesin heavy chain (KHC) at Ser314 stops motor activity [77]. Therefore, GSK3β plays a key role in regulating the universal transport of all synaptic vesicles, including HTT-containing vesicles within axons. Our results also reveal that while HTT-containing membranes contain active GSK3β and motor proteins, consistent with our previous observations [85, 87], increased levels of both active GSK3β and motor proteins were present on membranes enriched from HD patient-iNeurons (Figs. 1 and 2), likely contributing to the disruption of cargo motility. Furthermore, inhibition of GSK3β caused both CSP- and HTT-containing axonal blockages in larval nerves expressing non-pathogenic HTT (Fig. 4G), similar to the disruption of the motility of dense core vesicles [42]. Moreover, loss or excess GSK3β also caused CSP-containing axonal blockages [86, 87], indicating GSK3β is a universal regulator of axonal motility. Therefore, changes in GSK3β activity seen in HD could likely be responsible for defective axonal transport, which is proposed to be an early event in HD pathogenesis [72].

In contrast to GSK3β, surprisingly, we found that ERK has no direct role during axonal transport. Partial loss or excess of *rl*, the *Drosophila* homolog of ERK, did not cause axonal blockages (Fig. 5). Excess constitutively active ERK also failed to cause axonal transport defects (Fig. 5). Furthermore, inhibition of ERK in the context of larvae expressing non-pathogenic HTT only caused HTT-containing axonal blockages, but not CSP-containing blockages (Fig. 6). Taken together, ERK is likely only involved in regulating the transport of HTT and does not play a broader regulatory role in the motility of other axonal cargo. While ERK activity has been shown to be controlled by several different mechanisms, including scaffolding and anchoring proteins, dictating when and where ERK is activated and limiting ERK localization to specific subcellular compartments [109], one prediction is that ERK phosphorylation of HTT could influence HTT’s ability to associate with membranes during initial assembly onto axonal cargoes, a process thought to occur at cell bodies and/or at terminals prior to long-distance transport. Here, we anticipate that ERK and HTT would only predominantly associate within cell bodies/terminals. Indeed, ERK localizes to synapses [110–113] and has been reported to modulate synaptic events such as functional synapse formation [14], vesicle exocytosis [114], synaptic vesicle mobilization and release [115], glutamate neurotransmitter release [116, 117] and synaptic vesicle trafficking [118]. Intriguingly, in vitro work also demonstrated that ERK phosphorylates dynamin to inhibit dynamin-microtubule interactions [118]. Perhaps there is a putative endocytic pathway at synapses involving crosstalk between ERK-mediated events and HTT, which are known to affect synaptic vesicle recycling [29], likely through associations with HIP1 [41], AP-2 [119], and HIP1R [120] to facilitate clathrin-dependent endocytosis. Notably, clathrin-dependent endocytosis can drive the internalization of neurotrophic receptors such as TrkB [121] and EGFR [122], which both lead to the activation of ERK1/2-mediated signaling [122–125]. It is also possible that ERK phosphorylation of HTT affects its association with receptors, synaptic proteins, and/or endocytosis-related proteins. Future work will be needed to test these predictions.

### ERK dampens pathogenic HTT-mediated apoptotic pathways

ERK signaling was shown to be deficient in striatal neurons of multiple transgenic HD mice [126, 127]. Interestingly, either expression of constitutively active ERK [126] or pharmacological stimulation of ERK activity via RB5 [127] or Fisetin [88] resulted in decreased cell death in HD mice. Similarly, we also found that changing the dose of ERK modulated HTT accumulations and cell death caused by pathogenic HTT. While pharmacological inhibition of ERK increased HTT accumulations and cell death in larval brains expressing pathogenic HTT (Fig. 6), adding ERK back with excess pathogenic HTT rescued these phenotypes (Fig. 7). Therefore, ERK signaling may play a compensatory role in preventing neuronal cell death elicited by neuronal dysfunction in HD [89]. We propose that ERK’s protective effect is primarily a response to the cellular defects caused by pathogenic HTT as opposed to being directly involved in pathways contributing to HD pathogenesis. However, since we discovered that ERK1 is capable of associating with and phosphorylating normal and pathogenic HTT, it is possible that ERK also plays a more direct role in HD pathogenesis than previously thought.

Neuronal survival hinges on the uptake of anti-apoptotic extrinsic factors known as neurotrophins, such as BDNF and NGF. Upon activation of their respective receptor tyrosine kinases, these factors undergo internalization into signaling endosomes, which are then transported to the cell body via dynein-mediated movement. Neurotrophin signaling fosters neuronal survival by initiating the ERK1/2 pathway [128]. Notably, active ERK1/2 can also facilitate the assembly of signaling endosomes by phosphorylating cytoplasmic dynein and promoting its association with RAB7-positive signaling endosomes [94]. Indeed, while disrupted neurotrophic signaling is well-documented in HD models [31, 129], we previously showed that HTT is present on retrogradely moving RAB7-containing signaling endosomes that likely contain BDNF and BMP [34]. Perhaps ERK-mediated phosphorylation of HTT influences the assembly of signaling endosomes. Indeed, ERK1/2 inhibition caused HTT cargo accumulations within axons but did not affect other synaptic cargo labeled by CSP (Fig. 6). Therefore, ERK1/2 may influence pathways involving only a subset of axonal cargoes that affect neuronal survival, such as HTT-Rab7-containing signaling endosomes. Alternatively, since ERK phosphorylates the intermediate chain of dynein [94], which can bind HTT [48, 49], perhaps ERK phosphorylation may also promote HTT-dynein associations, similar to how AKT1 phosphorylation promotes HTT associations with kinesin-1 [38]. Accordingly, we predict that ERK may play a role in the assembly of signaling endosomes by phosphorylating HTT and dynein. While further study is needed to test the predictions of this proposal, our findings underscore the potential of targeting the ERK1/2 pathway for therapeutic intervention in mitigating polyQ-mediated neuronal cell death.

Alternatively, since the caspase cleaved 587-3144 C-terminal fragment containing the three putative ERK phosphorylation sites was found to be toxic [130], with the C-terminus interacting with the N-terminal fragment begs the question whether ERK-mediated events on HTT are independent of polyQ-expansion. A shorter C-terminal HTT fragment (aa2568-3144) containing two ERK putative phosphorylation sites was also shown to be toxic [131]. Therefore, C-terminus-dependent mechanisms that are mediated by ERK phosphorylation could contribute to HD pathogenesis. While the HTT C-terminal mechanisms are still ambiguous, several unanswered questions remain. Do ERK-mediated phosphorylation/dephosphorylation events cause conformational changes to full-length HTT or to the caspase-cleaved HTT C-terminal fragments for ON/OFF states? Does ERK phosphorylation at the C-terminus structurally alter HTT’s binding properties with membranes or dictate the recruitment of proteins that structurally re-organize and aid HTT’s scaffolding role at membranes? Further studies would be needed to test these predictions and to isolate the role of caspase-cleaved HTT fragments that are likely to be phosphorylated by GSK3β and ERK.

## Materials and methods

### Neuronal culture, characterizations, and biochemical assays

#### WT and HD iPSC cultures and neuronal differentiation

The following cell lines were obtained from the NIGMS Human Genetic Cell Repository at the Coriell Institute for Medical Research: iPSCs from WT (ND38555-polyQ = 17, 48 y, female) and HD (ND42222-polyQ = 109, 9 y, female) patients were purchased from the NINDS Repository. iPSCs were grown and expanded on corning matrigel (Fisher) using E8 iPSC media (Invitrogen). Pluripotency was analyzed using an antibody against OCT-3/4 (Santa Cruz, 1:200), and Hoechst was used as a nuclear staining, as detailed below. After 4 passages iPSCs were differentiated into neuronal precursors (NPCs) using PSC neural induction media (Invitrogen) and published protocols (publication #MAN0008031). NPCs were identified using an antibody against Nestin (Santa Cruz, 1:200) and then differentiated into mature iNeurons using neurobasal media supplemented with 1× B27 and 2 mM glutamine (Invitrogen, ThermoFisher). Differentiated neurons, identified using antibodies against MAP2 (BD Biosciences, 1:100), βIII-tubulin (Biolegend, 1:100) and Synaptophysin (Phosphosolutions, 1:100) exhibited an extensive neurite network after 21 days at which time they were used for electrophysiology experiments, biochemical experiments, immunofluorescence, and transfections.

#### Electrophysiology in iNeurons

To study the functionality of the differentiated neurons, cells were selected for whole-cell patch-clamp electrophysiology as previously done [33]. In all experiments, cells were bathed in artificial cerebrospinal fluid containing (in mM): 124 NaCl, 2.5 KCl, 2.5 CaCl_2_, 2 MgSO_4_, 1.25 KH_2_PO_4_, 26 NaHCO_3_, 10 glucose, 4 sucrose, pH 7.2 (NaOH), 324 mOsm. ACSF was prepared fresh daily.

#### Voltage-clamp experiments

To reveal currents through voltage-gated Na+ channels, borosilicate glass pipettes (8—12 MΩ) were filled with intracellular solution containing (in mM): 117 CsMeSO4, 5 tetraethylammonium (TEA), 2 MgATP, 0.3 Na2GTP, 20 HEPES, 0.4 EGTA, 2.8 NaCl, pH 7.2 (CsOH), 263 mOsm. To reveal currents through voltage-gated K+ channels, pipettes contained (in mM): 120 K-gluconate, 20 KCl, 10 HEPES, 0.2 EGTA, 2 MgCl2, 2 MgATP, 0.3 Na2GTP. To verify the identity of the currents, suspect Na+ channel currents were tested for inhibition due to 1 µM tetrodotoxin (TTX) bath applied while suspect K+ channel currents were tested for inhibition due to 50 mM TEA bath applied. Currents elicited in the presence of an inhibitor were normalized to the maximum current elicited in the absence of an inhibitor. Currents were low-pass filtered (2 kHz, 4-pole Bessel, Axon Instruments 200B) and sampled (10 kHz, Molecular Devices, Digidata 1440A).

#### Current-clamp experiments

To elicit action potential waveforms, borosilicate glass pipettes (8—12 MΩ) contained the K + -based intracellular solution described above. Upon break-in, cells were held at −70 mV. Action potentials were elicited upon increasing the current injection in 10 pA increments. Membrane potentials were low-pass filtered (2 kHz, 4-pole Bessel, Multiclamp 700B) and sampled (10 kHz, Molecular Devices Digidata 1550B). All files were analyzed using Clampfit 10.5 (Molecular Devices).

#### Immunocytochemistry

human iPSCs/NPCs/iNeurons: WT and HD iPSC/NPC colonies or 21-day-old, differentiated neurons were washed in PBS pH 7.2 and fixed in 4% paraformaldehyde. A blocking solution (1× PBST with 5% BSA) was added to cells for 60 min prior to antibody incubation. Cells were incubated in primary antibodies, OCT-4 (Santa Cruz 1:100), Nestin (Santa Cruz 1:100), MAP2 (BD Biosciences 1:100) βIII-tubulin (Biolegend 1:100), or Synaptophysin (Phosphosolutions 1:100) for 16 h at 4 °C and appropriate secondary antibodies (AlexaFluor® 488 or 568, ThermoFisher) for 1 h at 25 °C. DAPI was used to stain nuclei. Cells were then imaged at 20×–40× (for iPSC and NPC) or 60×–100× (iNeurons). iNeurons were imaged on glass slide-bottom dishes (In Vitro Scientific China, D29–14-1-N). Fixed images were taken at 20x-100x using DAPI, FITC, TxRed, and Cy5 filters using a NikonTE-2000E inverted fluorescence microscope with a beam splitter containing narrow single-band GFP/DsRED filters and a Cool Snap HQ cooled CCD camera which were merged into a single RGB image to analyze colocalization noted as yellow or white puncta.

#### Preparation of protein extracts from human iNeurons

WT and HD iNeurons were manually removed from 6-well or 12-well plates using ice-cold homogenization buffer (10 mM HEPES, pH 7.4, 100 mM K acetate, 150 mM sucrose, 5 mM EGTA, 3 mM Mg acetate, 1 mM DTT) containing a cocktail of protease inhibitors (Roche) and 5 mM EDTA, blended for 30 s on ice using a motorized pestle, and then quickly snap-frozen in liquid nitrogen. Neuronal extracts were centrifuged at 1000 × *g* for 15 mins, and the supernatant was analyzed using western blot.

#### Sucrose gradient fractionations

PNS samples human iNeuron extracts were further fractionated into soluble fractions (SF), heavy membrane pellet (P1), and LM fractions by sucrose gradient ultra-centrifugations using lysis buffer (4 mM HEPES, 320 mM sucrose pH 7.4) containing a phosphatase and protease inhibitor cocktail (Pierce) as previously done [33, 34, 85, 87]. Briefly, 300 µl of PNS was combined with 300 µl 62% sucrose and layered onto a sucrose gradient (35% and 8% sucrose), and centrifuged at ~100,000 × *g* for 90 mins. The LM fraction (35/8 layer), the SF, and the heavy membrane pellet (P1) were removed and used in western blot analysis. 100 µl of lysis buffer was used to dissolve the heavy membrane pellet (P1).

#### Co-immunoprecipitation analysis

HTT-containing vesicles were isolated as described previously [33, 34]. In brief, 10ug of mouse monoclonal antibody to HTT (EMD Millipore, 1:250) were crosslinked with magnetic beads (Pierce Protein A/G Magnetic Beads) with rotation for 30 min using Pierce™ Crosslink Magnetic IP/Co-IP Kit with 0.25 mM DSS. 1000 μg of total protein from the human iNeuron LMs were incubated with the HTT-antibody crosslinked Magnetic Beads for 16 h at 4 °C with rotation and eluted with Pierce elution buffer (0.1 M Glycine, pH 2.8). Eluents were then either separated by SDS-PAGE and analyzed via western blot analysis as detailed below and/or stored in liquid nitrogen for Trypsin-digestion and LC-MS analysis as detailed below. Isolation of HTT vesicles using crosslinked magnetic beads occurred in the absence of detergents to preserve vesicular membranes.

#### SDS-PAGE and western blot analysis

Human iNeuron fractions in NuPage LDS sample buffer with 4 mM β-mercaptoethanol were run on 4–12% Bis-Tris gels (Invitrogen) and transferred to nitrocellulose membranes. Alternatively, human iNeurons HTT-IP samples were run on a Zn^2+^-phos-tag™ AAL-107 gel as per manufacturer’s recommended protocol (FujiFilm-Wako) using 50 µM AAL-107 solution. For the enhanced resolution of full-length HTT (350 kDa), a control gel with a ladder ran simultaneously beyond the 62 kDa standard “running-off” the gel. Similarly, phos-tag gels were transferred to nitrocellulose membranes. Blots were blocked using TBST with 5% BSA for 60 mins at 25 °C and incubated with primary antibodies (SYT1 (Thermofisher 1:1000), Rab4 (Abcam 1:1000), Rab5 (Abcam 1:1000), Rab2 (SCBT 1:500), Rab7 (SCBT 1:500), VPS35 (SCBT 1:500), SUMO2/3 (Cytoskeleton 1:500), KIF5A (Goldstein 1:250), KIF5B (Goldstein 1:250), KIF5C (Goldstein 1:250), DIC (Abcam 1:1000), DNCT (Abcam 1:1000), Actin (ThermoFisher 1:1000), Tubulin (Abcam 1:2000), HTT rabbit polyclonal (Abcam 1:1000), HTT mouse monoclonal (EMD Millipore 1:1000), Golgi (Millipore Sigma 1:1000), Cytochrome C (Santa Cruz 1:1000), TOM20 (CellSignaling Technology 1:500), MAP1B (SCBT 1:1000), MAP2 (BD Pharmigen 1:1000), Total AKT1 (CellSignaling Technology 1:1000), pAKT1 (Ser473, CellSignaling Technology 1:1000), Total GSK3α/β (CellSignaling Technology 1:1000), pGSK3α/β (pY279/pY216; Abcam 1:1000), or ERK (pan-ERK; BD Transduction Laboratories 1:1000) for 16 h at 4 °C. Blots were then incubated with anti-mouse or anti-rabbit secondary HRP-conjugated antibodies (ThermoFisher 1:1000) and imaged using a BioRad Chemi-doc system with Pierce ECL or diluted Femto substrate (1:5 in TBS). Images from three blots were quantified using ImageLab.

#### In vitro phosphorylation kinase assay

Recombinant GST-GSK3β or GST-ERK1 (SignalChem) were used for in vitro kinase assays. Potential substrates (HTT-IP) were incubated with 50 ng GSK3β or 50 ng ERK1 and 1 mCi/100 γ32P-ATP for 30 min at 37°C. The reaction was terminated using 4× sample buffer. Control reactions containing 30 µM of GSK3β inhibitor CHIR99021 (Selleck Chemical) and/or 30 µM ERK1 inhibitor SCH772984 (Selleck Chemical) were done to evaluate the specificity of the GSK3β or ERK1 phosphorylation assay. Proteins were separated by SDS-PAGE, and the gel was dried and sealed in saran wrap and exposed to X-ray film overnight. After exposure, gels were stained with Coomassie brilliant blue to visualize proteins.

#### Statistical analysis of immunoblotting assays

The statistical analysis used for each experiment is indicated in each figure legend. First, power and sample size (*n*) calculations were performed for each experimental paradigm: comparing two means from two samples, with two-sided equality to identify the sample size that corresponds to *α* = 0.05. For each experiment, a significance threshold of *p* < 0.05 (95% confidence) was used. Based on the power analysis for western blot assays, three separate membranes from three independent experiments were analyzed using ImageJ software. Individual data points for each analysis were averaged for each n and compared. Unless otherwise specified, the data compared was found to be normally distributed. Statistical significance of normal distributions was calculated by a two-sample two-tailed Student’s *t* test and/or ANOVA test in Excel, Minitab18 and/or by using the Proc GLM procedure followed by the pdiff mean comparison option in SAS Studio 3.81. Overlaid dot plots were constructed for all figures using OriginLab/OriginPro to represent mean ± SEM.

### Proteomics assays and analyses

#### Protein preparation and digestion

HTT-IP or total LM samples were dissolved in a detergent-containing buffer (50 mM Tris-formic acid (FA), 150 mM NaCl, 1% sodium deoxycholate, 2% sodium dodecyl sulfate (SDS), 2% IGEPAL® CA-630, pH 8.0) plus the protease and phosphatase inhibitor tablets (Roche Applied Science, Indianapolis, IN), and were sonicated for 30 s (non-continuously, 5 s as a burst) using a high-energy sonicator (Qsonica, Newtown, CT). The solution was then centrifuged at 20,000 × *g*, 4 °C for 30 min, and the supernatant portion was carefully transferred to Eppendorf tubes. Protein concentration was measured by bicinchoninic acid assay (BCA) kit (Pierce Biotechnology, Inc., Rockford, IL). One hundred μg of extracted proteins from every sample were utilized for LC-MS analysis. Reduction and alkylation of proteins were achieved by 30 min incubation with 3 mM tris (2-caboxyl) phosphine (TCEP) and 30 min incubation with 20 mM iodoacetamide (IAM), respectively. Both steps were conducted under 37 °C in darkness with constant vortexing in an Eppendorf Thermomixer® (Eppendorf, Hauppauge, NY). The proteins were then subjected to a unique surfactant-aided precipitation/on-pellet digestion (SOD) procedure. Precipitation of proteins was performed by stepwise addition of 9 volumes of cold acetone with continuous vortexing and incubation at −20 °C overnight. After centrifugation at 20,000 × *g*, 4 °C for 30 min, the supernatant (containing undesirable constituents which may severely impair the overall quality of MS analysis in the protein mixture, e.g. detergents, components of a cellular matrix) was removed, and the pellets were rinsed with 800 μl of cold acetone/water mixture (85/15, v/v %) and air-dried. Two phases of enzyme addition were employed for the on-pellet-digestion. In phase 1 (pellet-dissolving phase), 50 µL of Tris buffer (50 mM, pH 8.5) containing trypsin at an enzyme/substrate ratio of 1:40 (w/w) was added to the pellets and incubated at 37 °C for 6 h in an Eppendorf Thermomixer®; in phase 2 (complete-cleavage phase), another 50 µL of trypsin solution was added at an enzyme/substrate ratio of 1:40 (w/w). Then, the mixture was incubated at 37 °C overnight (12–16 h) to achieve complete digestion, and digestion was terminated by the addition of 1 μl formic acid. Supernatant for individual samples containing tryptic peptides derived from 6 μg of proteins was used for LC-MS analysis.

#### Long gradient nano-reverse-phase liquid chromatography/mass spectrometry (LC-MS)

An optimized gradient was utilized to resolve the complex peptide mixture, encompassing the following steps: 3 to 8% B over 15 min; 8 to 24% B over 215 min; 24 to 38% B over 115 min; 38–63% B over 55 min; 63 to 97% B in 5 min, and finally isocratic at 97% B for 15 min. Under such chromatographic settings, a peptide elution window of >345 min was achieved, with an average peak of <30 s and a peak capacity of >580.

An LTQ/Orbitrap-ETD hybrid mass spectrometer (Thermo Fisher Scientific, San Jose, CA) was employed to analyze the identity of peptides in the mixture. An “overfilling” approach, which allowed the reinforcement of MS sensitivity while simultaneously guaranteeing accuracy and resolution, was applied for peptide detection. The spray tip was rinsed by dripping 50% methanol after every three runs to keep ionization efficiency stabilized. The instrument was operated in the data-dependent product ion mode. One scan cycle included one MS1 scan (m/z 310–2000) in the profile mode at a resolution of 60,000 followed by seven MS2 scans in collision-induced dissociation (CID) activation mode to fragment the seven most abundant precursor ions identified in the MS1 spectrum. The target value for MS1 by Orbitrap was 8 × 106, under which the Orbitrap was meticulously calibrated for mass accuracy and Fourier transform (FT) transmission. The use of a high target valve on the Orbitrap enabled ultra-sensitive detection with no compromise to the mass accuracy and resolution. The dynamic exclusion was enabled with the following settings: repeat count = 1; repeat duration = 30 s; exclusion list size = 500; and exclusion duration = 40 s. The activation time was 30 ms, the isolation width was 3 Da for the linear ion trap (LTQ), the normalized activation energy was 35%, and the activation q was 0.25. Three biological replicates from each biological group (db/db versus db/+) were analyzed in a random manner.

#### Protein identification and quantification

Raw files were imported into PD, and DTA files were generated from MS2 spectra. The search parameters used were as follows: 25-ppm tolerance for precursor ion mass and 1.0 Da for fragment ion mass. Two missed cleavages were permitted for tryptic peptides. Carbamidomethylation of cysteines and oxidation of methionine were set as fixed and variable modification, respectively. The false discovery rate (FDR) was detected by the usage of a target-decoy search strategy, in which the sequence database contains each sequence in both forward and reversed orientations and enables the estimation of FDR. Scaffold software (v4.3.2, Proteome Software, Portland, OR) was used to validate MS/MS-based peptide and protein identification based on cross-correlation (Xcorr) and Delta Cn values. The peptide filtering criteria included Delta Cn scores >0.1 and Xcorr scores >1.1, 1.4, 1.7, and 2.5 for singly, doubly, triply, and quadruply charged peptides. Stringent cutoffs for the Delta Cn and Xcorrs scores, plus the additional requirement that at least two distinct peptide sequences are needed for the identification of a protein, resulting in a considerably low FDR (0.19% at the peptide level). Shared peptides are retained on the identification level but are further evaluated on the quantification level for the congruity of including these peptides for quantification.

SIEVE® software (v2.1.377, Thermo Scientific, San Jose, CA) was used to perform chromatographic alignment and global intensity-based MS1 feature detection/extraction, consisting of: 1) Global chromatographic alignment of LC-MS runs via the application of ChromAlign algorithm. The alignment scores given by SIEVE, as well as the intensities of base-peak-ion current, were monitored and benchmarked for quality control; 2) Determination of quantitative “frames” based on mass-to-charge (m/z) and retention time in the aligned dataset. Only frames with high-quality area under the curve (AUC) with signal-to-noise ratio >10 were picked so that the quantitative reliability was assured; 3) Calculation of ion intensities among all “frames”. The output files were then merged with the spectrum report file exported from Scaffold to link the MS2 fragmentation scans with each “frame” using an in-house developed R package, IonStarStat (available at https://github.com/shxm725/IonStarstat). The normalization of ion current intensities, the rejection of outlier peptides with aberrant intensities, and the aggregation of sum ion intensities from “frame” level to the protein level were also achieved by IonStarStat. The expression ratio for each protein was calculated based on the ion current peak areas of three replicates in control/WT and HD groups.

#### Exclusion criteria for raw LC-MS data from WT and HD LMs

From 3 independent biological replicates, proteins were identified using UNIPROT accession IDs from the *Homo sapiens* proteome FASTA database. 3931 unique proteins from WT LMs and 3074 unique proteins from HD LMs were identified from LC-MS across 3 independent biological replicates. After excluding proteins that were present in less than 2 independent trials (< 2) and excluding proteins with less than three unique peptides (spectral count (SpC) < 3) per trial, 3046 proteins remained in the WT LM data, and 2127 proteins remained in the HD LM data. Of these, 1998 proteins were present in both WT and HD LM samples, 1048 proteins were only in WT LMs, and 129 proteins were only in HD LMs.

#### Exclusion criteria for raw LC-MS data from WT and HD HTT-IPs

From 3 independent biological replicates, proteins were identified using UNIPROT protein accession IDs from the *Homo sapiens* proteome FASTA database. 2332 unique proteins from WT HTT-IPs and 2308 unique proteins from HD HTT-IPs were identified from LC-MS across 3 independent biological replicates. In addition to excluding proteins that were present in less than 2 independent trials (<2) and excluding proteins with less than three unique peptides (spectral count (SpC) < 3) per trial, the SpC of a protein in WT or HD HTT-IP was deducted from a negative control-IP in which WT or HD LMs were incubated with magnetic beads without antibody. Following these criteria, 1062 proteins remained in the WT HTT-IP data, and 1639 proteins remained in the HD HTT-IP data. Of these, 800 proteins were present in both WT and HD HTT-IPs samples, 87 proteins were only in WT HTT-IPs, and 714 proteins were only in HD HTT-IPs.

#### Statistical analysis of HTT-IP and LM LC-MS data

First power and sample size (*n*) calculations were performed on Minitab18 for each experimental paradigm: comparing 2 means from 2 samples, with two-sided equality to identify the sample size that corresponds to *α* = 0.05. Statistical analysis used for each experiment is indicated in each figure legend. SpC’s of identified proteins from total LMs and HTT-IPs were analyzed using three biological replicates. SpC’s of identified proteins from HTT-IPs were first normalized to the HTT SpC within each trial to account for any difference in the amount of HTT IP’d between replicates and then analyzed using three biological replicates. Statistical significance was calculated by one-way ANOVA/post-hoc analysis to reduce Type I error, followed by Welch’s *t* tests to test to compare individual groups in Excel and Minitab18. Statistical analysis reported in figures report results from Welch’s *t* tests, as results from ANOVA/post-hoc and Welch’s *t* tests were consistent. Fold change (FC) between WT and HD samples was calculated by dividing the HD SpC of an individual protein by its respective WT SpC and normalizing to the total SpC from each sample. To reduce the false discovery of identified hits, an effect size cut-off for FC was set to log2FC > |1| (±2X) as in [132] and as done previously in [133]. This approach has also been applied in previous comparative proteomics studies focused on HTT [12, 134].

#### Gene ontology (GO) analysis

Functional enrichment analysis for biological processes, molecular functions, and cellular components of lists of proteins that were increased, decreased, gained, or lost with either HTT-IP or LMs between WT and HD iNeurons were analyzed using the g:Profiler resource (http://biit.cs.ut.ee/gprofiler/gost) and cross-referenced with the Database for Annotation, Visualization and Integrated Discovery (DAVID) Bioinformatics Resource v6.7 (http://david.abcc.ncifcrf.gov). Cellular components assigned by g:Profiler were manually examined, ranked by p-value as well as coverage (%gene intersection), and then distributed into selected categories (Fig. 3.1, 3.2). Furthermore, reactome pathways were analyzed using the g:Profiler resource (http://biit.cs.ut.ee/gprofiler/gost) and cross-referenced with GeneCodis4.0 [135]. Enriched reactome pathways assigned by g:Profiler were manually examined, ranked by *p* value and coverage (% intersection).

#### Kinome enrichment analysis

Details about kinase enrichment and signaling pathways were analyzed from lists of proteins that were increased, decreased, gained, or lost with either HTT-IP or LMs between WT and HD iNeurons using the Kinase Enrichment Analysis v.3 (KEA3; [136]) from the Icahn School of Medicine at Mount Sinai (https://maayanlab.cloud/kea3/) and/or the Kinome Mapping Hub Resource through Cell Signaling Technology, Inc (http://www.kinhub.org/kinmap/).

#### Data visualization software

lists of proteins that were increased, decreased, gained, or lost with either HTT-IP or LMs between WT and HD iNeurons were used to generate heatmaps through the Morpheus software by the Broad Institute (https://software.broadinstitute.org/morpheus/) and volcano plots were generated through OriginLab/OriginPro and/or the VolcaNoseR data visualization software (https://huygens.science.uva.nl/) through Rstudio.

### Drosophila studies

#### Drosophila genetics

Pan-neuronal driver APPL-GAL4 was used for neuronal expression of transgenic lines [137–139]. Unless stated, fly stock were obtained from BDSC. In brief, males from UAS-HTTex1.Q25-eGFP (gift from Norbert Perrimon; [139]), UAS-HTTex1.Q103-eGFP (gift from Norbert Perrimon; [139]), UAS-sgg.B (sgg^OE^), UAS-sgg.S9A (sgg^active^), UAS-sgg.A81T (sgg^DN^), UAS-rl.K (rl^OE^), UAS-rl^SEM^ (rl^active^), or rl^1^ (rl^LoF^) were crossed to APPL-GAL4 virgins. UAS-HTTex1.Q103-eGFP or UAS-HTTex1.Q25-eGFP males were crossed to APPL-GAL4;T(2:3),CyO,TM6B,Tb/Pin88K virgin females. The chromosome carrying T(2:3),CyO,TM6B,Tb is referred to as B3 and carries the dominant markers, Hu, Tb and CyO. The larval Tb (Tubby) marker is used to select larvae of interest. APPL-GAL4/Y;UAS- HTTex1.Q103-eGFP or UAS-HTTex1.Q25-eGFP/Cyo,TM6B males were then crossed with UAS-rl.K (rl^OE^) virgin females. In all cases, female 3rd instar larvae were selected. Crosses were maintained at 29 °C for protein overexpression and controlled for humidity (60%).

#### Larval feeding and incubations

For chemical feeding experiments, 3rd instar larvae expressing UAS-HTTex1.Q25-eGFP, UAS-HTTex1.Q103-eGFP, or UAS-HTTex1.Q103-eGFP;UAS-r.K larvae were grown in fly flood containing buffer (0.1%DMSO), 10 µM CHIR99021 (Caymen Chemical) dissolved in buffer/0.1% DMSO [77, 85], or 10 µM SCH772984 (Caymen Chemical) dissolved in buffer/0.1%DMSO [78]. After 24 h, female larvae were selected for behavioral studies and/or dissections prior to fixation and imaging.

#### Larval preparations, immunohistochemistry, and quantifications

Third instar *Drosophila* larvae were dissected and fixed in 8% paraformaldehyde. DCSP-3 (DSHB, 1:10) antibody was used in conjunction with secondary antibodies anti-mouse or anti-rabbit AlexaFlour®488, AlexFlour®568, or Alexaflour®647 (ThermoFisher, 1:100). Images of segmental nerves were collected using a Nikon Eclipse TE 2000U microscope using the 40× or 60× objective (Nikon, Melville, NY, USA) alongside the FITC (488 nm), TxRED (568 nm), both FITC/TxRED (568/488 nm using a dual-view beam splitter attachment) filters. TxRED and FITC images were merged into a single RGB image to analyze colocalization noted as yellow puncta. Axonal blockages were quantified as previously done [30]. For each genotype, a minimum of 6 confocal optical images across six larvae were imaged. Subpixel imaging refers to the subpixel detection accuracy of fluorescent puncta, which was previously confirmed by directly comparing Gaussian fitting of conventional microscopy data analyzed to high-resolution imaging [109]. For NMJ analysis, HRP-FITC or HRP-TxRED (1:50, Jackson ImmunoResearch Labs). Non-tubby, female larvae were dissected, fixed, and stained with HRP. Quantification of NMJ morphology and HTT-eGFP intensity was performed as previously done [33, 79]. We examined type-1 synaptic boutons between muscles 6/7 at larval abdominal segments A4-A5 of third instar larval. Images of NMJs were collected using a Nikon Eclipse TE 2000 U microscope at ×60 (Nikon, Melville, NY, USA). For each genotype, a minimum of 12 optical images across 4–6 larvae were imaged. Total NMJ length (μm) was measured using NIH ImageJ software as done previously [33, 34].

#### TUNEL assay

Third instar larvae were dissected and fixed as described above prior to being permeabilized with 5% saponin for 20 min at 25 °C. TUNEL assay was performed on permeabilized larval brains using the In Situ Cell Death Detection Kit (Roche) per manufacturer’s instructions. Incubation in DNAse I was used as a positive control. The number of puncta in each brain was quantified in ImageJ (NIH) using the Threshold tool and Analyze Particles tool. At least four adult brains were imaged and quantified from each genotype.

#### Quantification of *Drosophila* larval velocities

Larval velocity was performed by visualizing third instar larval crawling patterns on 1% agarose gel, dyed blue for added contrast, that was embedded with a 0.25 cm × 0.25 cm grid. Once placed at the center of the agarose gel, a 2-min interval recording began. Controlling for temperature (~25 °C) and humidity (~60%), twenty larvae were tested per condition across two independent trials. Quantifications of the larval velocity were measured using NIH ImageJ software and Track-mate. A total of 1.5 min of the 2-min recording was utilized for quantification purposes allotting the initial 15 s for larva self-adjustment after being placed in the center of the agarose gel.

#### Statistical analysis of *Drosophila* assays

The statistical analysis used for each experiment is indicated in each figure legend. First power and sample size (*n*) calculations were performed for each experimental paradigm: comparing 2 means from two samples, with two-sided equality to identify the sample size that corresponds to α = 0.05. For each experiment, a significance threshold of *p* < 0.05 (95% confidence) was used. Based on the power analysis, quantifications were performed across 4–6 larvae for *Drosophila* assays. The n-value refers to the number of larvae. Individual data points for each analysis were averaged for each n and compared. Unless otherwise specified, the data compared was found to be normally distributed. Statistical significance of normal distributions was calculated by a two-sample two-tailed Student’s *t* test and/or ANOVA test in Excel, Minitab18 and/or by using the Proc GLM procedure followed by the pdiff mean comparison option in SAS Studio 3.81. Overlaid dot plots were constructed for all figures using OriginLab/OriginPro to represent mean ± SEM.

**Table:** 

RESOURCE	SOURCE	IDENTIFIER
**Antibodies and dyes**		
Mouse anti-Nestin	Santa Cruz	Cat# sc-23927 RRID: AB_627994
Mouse anti-OCT-3/4 (C-10)	Santa Cruz	Cat# sc-5279 RRID: AB_628051
Mouse anti-MAP2	BD Biosciences	Cat# 801212 RRID: AB_2721321
Mouse anti-βIII-tubulin (TUBB3)	Biolegend	Cat# ab152 RRID: AB_390204
Mouse anti-SYP (SY38)	Phosphosolutions	Cat# MAB5258 RRID: AB_2313839
Rabbit anti-SYT1	Thermofisher	Cat# 1975-STG RRID: AB_2492251
Mouse anti-HTT (1HU-4C8)	EMD Millipore	Cat# MAB2166 RRID: AB_2123255
Rabbit anti-HTT (EP867Y)	Abcam	Cat# ab45169 RRID: AB_733062
Rabbit anti-RAB4 (monoclonal)	Abcam	Cat# ab109009 RRID: AB_10887396
Rabbit anti-RAB4 (polyclonal)	Abcam	Cat# ab13252 RRID: AB_2269374
Rabbit anti-RAB5	Abcam	Cat# ab18211 RRID: AB_470264
Mouse anti-RAB7	Santa Cruz Biotechnology	Cat# sc-13156 RRID: AB_627385
Mouse anti-RAB2	Santa Cruz Biotechnology	Cat# sc-133081 RRID: AB_2176892
Mouse anti-VPS35	Santa Cruz Biotechnology	Cat# sc-374372 RRID: AB_10988942
Mouse anti-SUMO2/3	Cytoskeleton, Inc.	Cat# ASM24 RRID: AB_2884969
Mouse anti-KIF5A	Laboratory of Lawrence Goldstein	[140]
Mouse anti-KIF5B	Laboratory of Lawrence Goldstein	[140]
Mouse anti-KIF5C	Laboratory of Lawrence Goldstein	[140]
Rabbit anti-DCTN1 (dynactin 1)	Abcam	Cat# ab96004 RRID: AB_10677601
Mouse anti-DYNC1I1 (74.1)	Abcam	Cat# ab23905 RRID: AB_2096669
Rabbit anti-ACTA1/Actin	ThermoFisher Scientific	Cat# MA5-32479 RRID: AB_2809756
Mouse anti- TUBA4A (tubulin, alpha 4A)	Abcam	Cat# ab7291 RRID: AB_2241126
Mouse anti-Golgi (7H6D7C2)	Millipore Sigma	Cat# 345867 RRID: AB_564660
Mouse anti-Cyt-c-p (A-8)	Santa Cruz Biotechnology	Cat# sc-13156 RRID: AB_627385
Rabbit anti-TOM20 (D8T4N)	Cell Signaling Technology	Cat# 42406S RRID: AB_2687663
Mouse anti-MAP1B	Santa Cruz Biotechnology	Cat# sc-365668 RRID: AB_10847224
Mouse anti-MAP2	BD Pharmigen	Cat# 556320 RRID: AB_396359
Rabbit anti-AKT1	Cell Signaling Technology	Cat# 75692 RRID:
Rabbit anti-pAKT1 (Ser473)	Cell Signaling Technology	Cat# 9018 RRID:
Mouse anti-tyrosine hydroxylase	EMD Millipore	Cat# ab152 RRID: AB_390204
Rabbit anti-LAMP1	Abcam	Cat# ab30687 RRID: AB_775973
Rabbit anti-GSK3α/β	Cell Signaling Technology	Cat# 5676 RRID: AB_10547140
Mouse anti-pGSK3α/β (pY279/pY216)	Abcam	Cat# ab68476 RRID: AB_10013745
Mouse anti-ERK (pan ERK)	BD Transduction Laboratories	Cat# 610124 RRID: AB_397529
Hoechst	Thermofisher	Cat# H3570 RRID: AB_10626776
Mouse anti-DCSP-3 (1G12)	Developmental Studies Hybridoma Bank	Cat# DCSP-3 (1G12) RRID: AB_528184
Anti-Mouse Alexa Fluor® 488	Invitrogen	Cat# A11001 RRID: AB_2534069
Anti-Mouse Alexa Fluor® 568	Invitrogen	Cat# A11004 RRID: AB_2534072
Anti-Mouse Alexa Fluor® 647	Invitrogen	Cat# A21235 RRID: AB_2535804
Anti-Rabbit Alexa Fluor® 488	Invitrogen	Cat# A11008 RRID: AB_143165
Anti-Rabbit Alexa Fluor® 568	Invitrogen	Cat# A11011 RRID: AB_143157
Anti-Rabbit Alexa Fluor® 647	Invitrogen	Cat# A21244 RRID: AB_141663
Anti-mouse secondary antibody, HRP	Invitrogen	Cat# 32430 RRID: AB_1185566
Anti-Rabbit secondary antibody, HRP	Invitrogen	Cat# 32460 RRID: AB_1185567
Alexa Fluor® 594 Goat Anti-Horseradish Peroxidase	Jackson Immuno Research Labs	Cat# 123-585-021 RRID: AB_2338966
Fluorescein (FITC) Goat Anti-Horseradish Peroxidase	Jackson Immuno Research Labs	Cat# 123-095-021 RRID: AB_2314647
**Experimental models: human cell lines**		
ND42222, polyQ=109, 9 y, female	NINDS Repository (Coriell Institute for Medical Research -Camden, NJ)	Cat# ND42222 RRID: CVCL_Y844
ND38555, polyQ=17, 48 y, female	NINDS Repository (Coriell Institute for Medical Research -Camden, NJ)	Cat# ND3855 RRID: CVCL_Y822
**Chemicals, peptides, recombinant proteins, and consumables**		
GSK3β inhibitor CHIR99021 (CT99021)	Selleck Chem	Cat# S1263 PubChem: 9956119
ERK1/2 inhibitor SCH772984	Selleck Chem	Cat# S7101 PubChem: 24866313
GST-GSK3β	Signal Chem	Cat# G09-10G RRID: N/A
GST-ERK1	Signal Chem	Cat# M29-10G RRID: N/A
Corning Matrigel	ThermoFisher Scientific	Cat# CB40230A RRID: N/A
Advanced DMEM/F12	Invitrogen	Cat# 12634028 RRID: N/A
Essential 8 media	Invitrogen	Cat# A1517001 RRID: N/A
Neurobasal media	Invitrogen	Cat# 21103049 RRID: N/A
PSC neural induction media	Invitrogen	Cat# A1647801 RRID: N/A
B27 supplement media	Invitrogen	Cat# 17504-044 RRID: N/A
Protease inhibitor cocktail	Pierce	Cat# PIA32965 RRID: N/A
Phosphatase inhibitor	Pierce	Cat# PI88667 RRID: N/A
Protein A/G Magnetic Beads	Pierce	Cat# PI88802 RRID: N/A
Vecta Shield Mounting Medium	Vector Laboratories	Cat# NC9265087 RRID: N/A
Phos-tag™ Acrylamide AAL-107	FujiFilm	Cat# 300-93523 RRID: N/A
In Situ Cell Death Detection Kit	Roche	Cat# 11684795910 RRID: N/A
DNase I	Roche	Cat# 11284932001 RRID: N/A
**Experimental Models:** ***D. melanogaster*** **organisms/strains**		
*P{Appl-GAL4.G1a}1,* *y*^*1*^ w*	Bloomington *Drosophila* Stock Center	BDSC: 32040; FlyBase: FBst0032040
*Appl-Gal4; T(2,3), CyO, TM6B,Ttb*^*1*^/*Pin*^*88k*^	Laboratory of Lawrence Goldstein	[137]
*pUAST-HTTex1-25Q-eGFP*	Laboratory of Norbert Perrimon	[139]
*pUAST-HTTex1-103Q-eGFP*	Laboratory of Norbert Perrimon	[139]
*w1118; P{UAS-sgg.B}MB5* (*sgg*^OE^)	Bloomington *Drosophila* Stock Center	BDSC: 5361 FlyBase: FBst0005361
*w1118; P{UAS-sgg.S9A}MB7/TM6C* (*sgg*^active^)	Bloomington *Drosophila* Stock Center	BDSC: 5362 FlyBase: FBst0005362
*w1118; P{UAS-sgg.A81T}MB2* (*sgg*^LoF^)	Bloomington *Drosophila* Stock Center	BDSC: 5359 FlyBase: FBst0005359
*w1118; P{UAS-rl.K}2A* (*rl*^OE^)	Bloomington *Drosophila* Stock Center	BDSC: 36270 FlyBase: FBst0036270
y1 w*; P{UAS-rl^*Sem*^*.S}2* (*rl*^active^)	Bloomington *Drosophila* Stock Center	BDSC: 59006 FlyBase: FBst0059006
*rl*^*1*^ (*rl*^LoF^)	Bloomington *Drosophila* Stock Center	BDSC: 386 FlyBase: FBst0000386
**Software/algorithms**		
MATLAB-based particle tracking program	Laboratory of Danuser	[141, 142]
ImageJ	Schneider et. al., 2012 https://imagej.net/	RRID: SCR_003070
Metamorph/Metavue Imaging Software	Molecular Devices, Sunnyvale, CA, USA	RRID: SCR_002368
Minitab18	https://www.minitab.com/en-us/	RRID: SCR_014483
Microsoft Excel	https://www.microsoft.com/en-gb/	RRID: SCR_016137
RStudio	http://www.rstudio.com/	RRID:SCR_000432
OriginLab/OriginPro	https://www.originlab.com/	RRID: SCR_014212

## Supplementary information

Figure S1

Figure S2

Figure S3

Figure S4

Figure S5

Figure S6

Figure S7

Table S1

Table S2

## Data Availability

All data generated or analyzed during this study are included in this published article or in the supplementary information. Raw data are available from the corresponding author upon request.
