# Supplementary figures and images for: Opposing roles for GSK3β and ERK1-dependent phosphorylation of huntingtin during neuronal dysfunction and cell death in Huntington’s disease
Source: Cell Death Dis. 2025 Apr 22;16(1):328. doi: 10.1038/s41419-025-07524-0 (PMC12015319; doi:10.1038/s41419-025-07524-0)

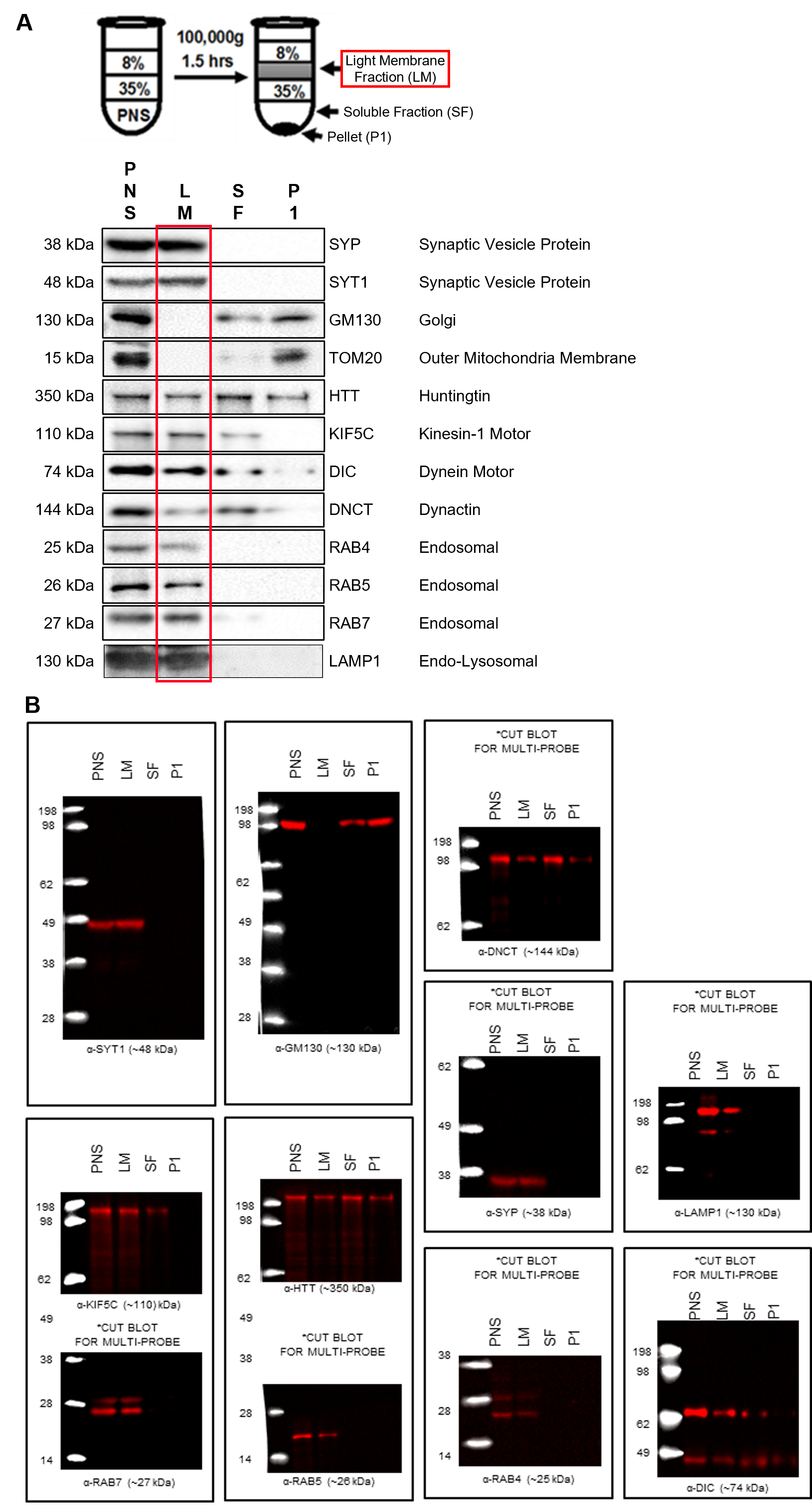

Supplement: Supplementary file 1 — Figure S1 [file 41419_2025_7524_MOESM1_ESM.png]

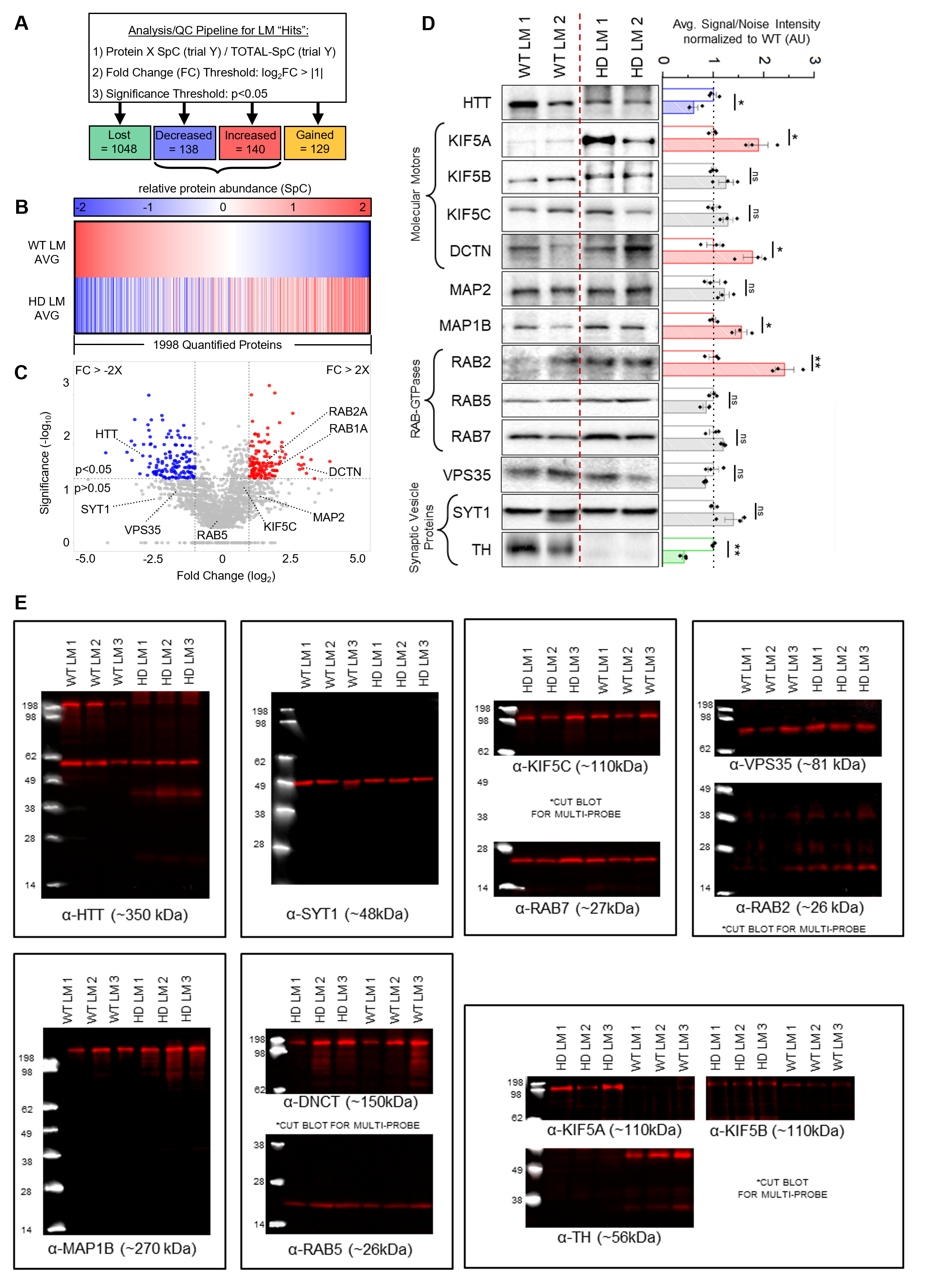

Supplement: Supplementary file 2 — Figure S2 [file 41419_2025_7524_MOESM2_ESM.png]

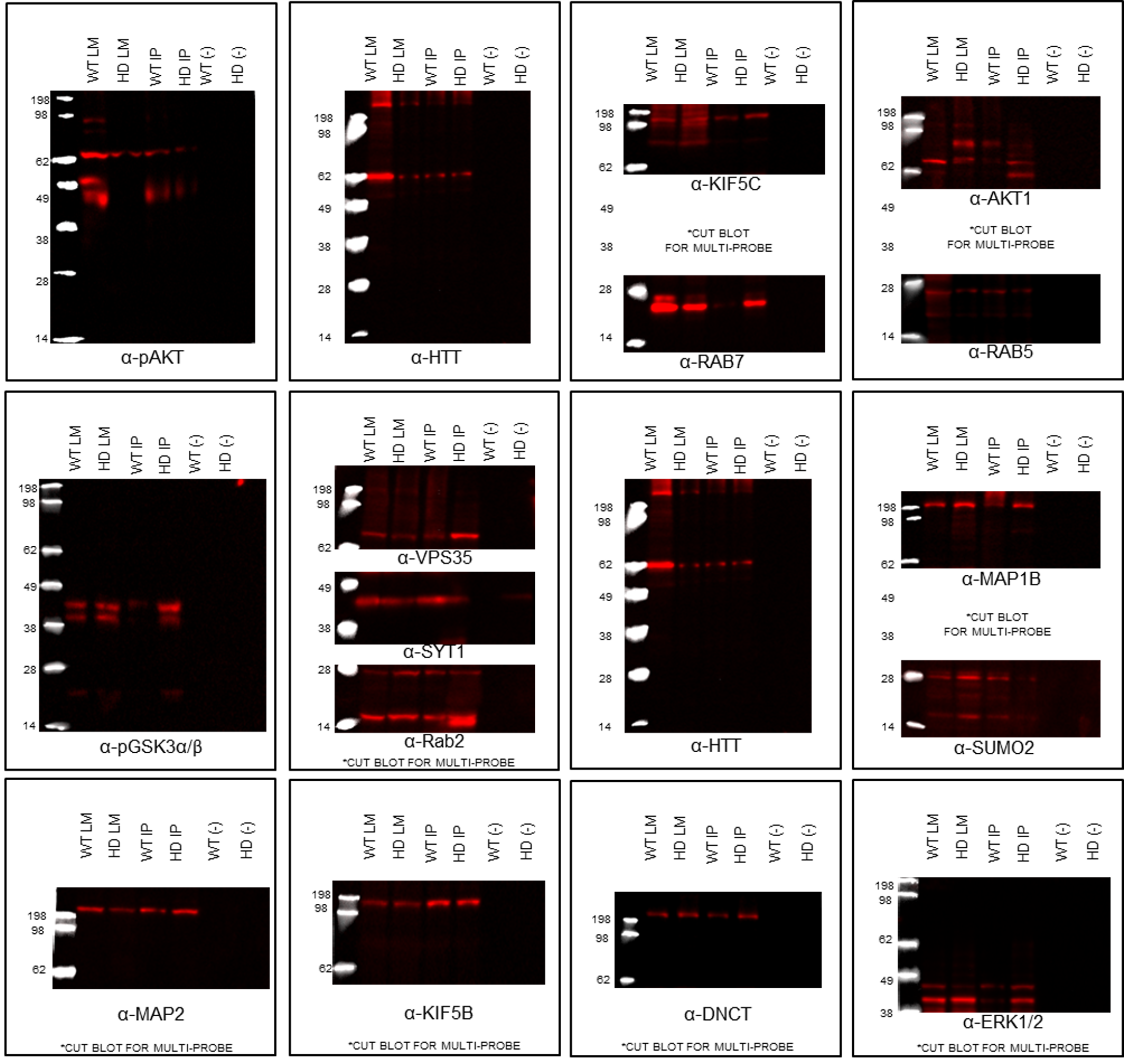

Supplement: Supplementary file 3 — Figure S3 [file 41419_2025_7524_MOESM3_ESM.png]

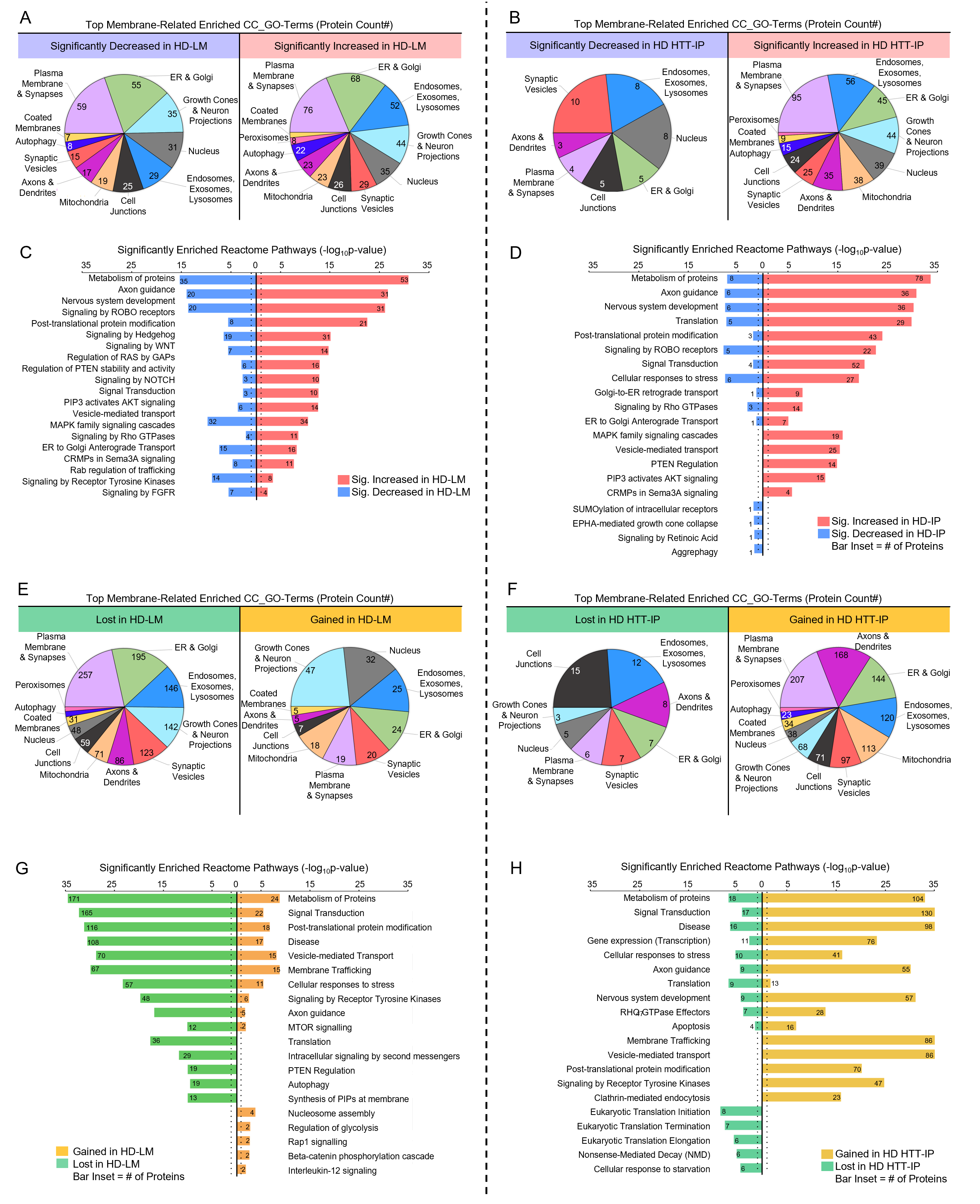

Supplement: Supplementary file 4 — Figure S4 [file 41419_2025_7524_MOESM4_ESM.png]

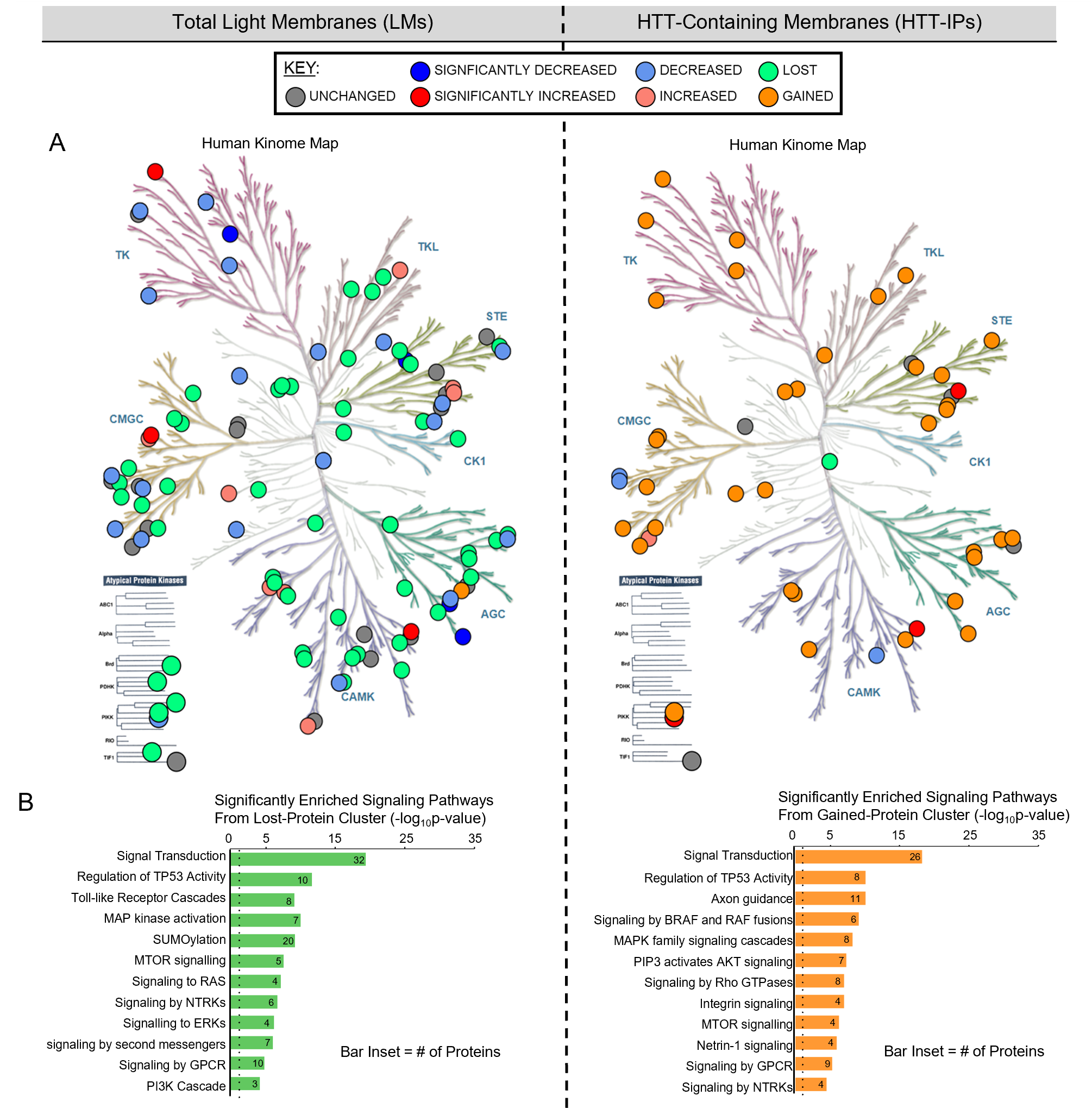

Supplement: Supplementary file 5 — Figure S5 [file 41419_2025_7524_MOESM5_ESM.png]

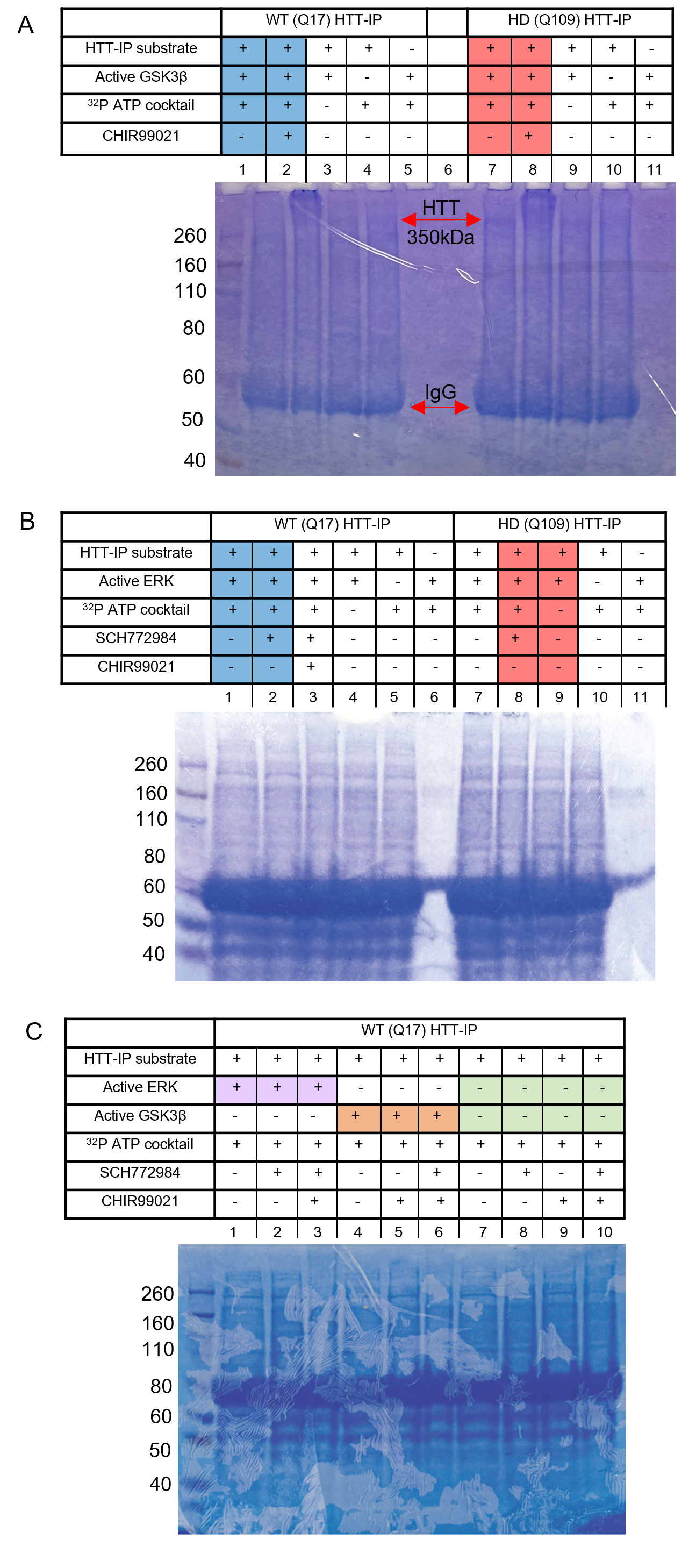

Supplement: Supplementary file 6 — Figure S6 [file 41419_2025_7524_MOESM6_ESM.png]

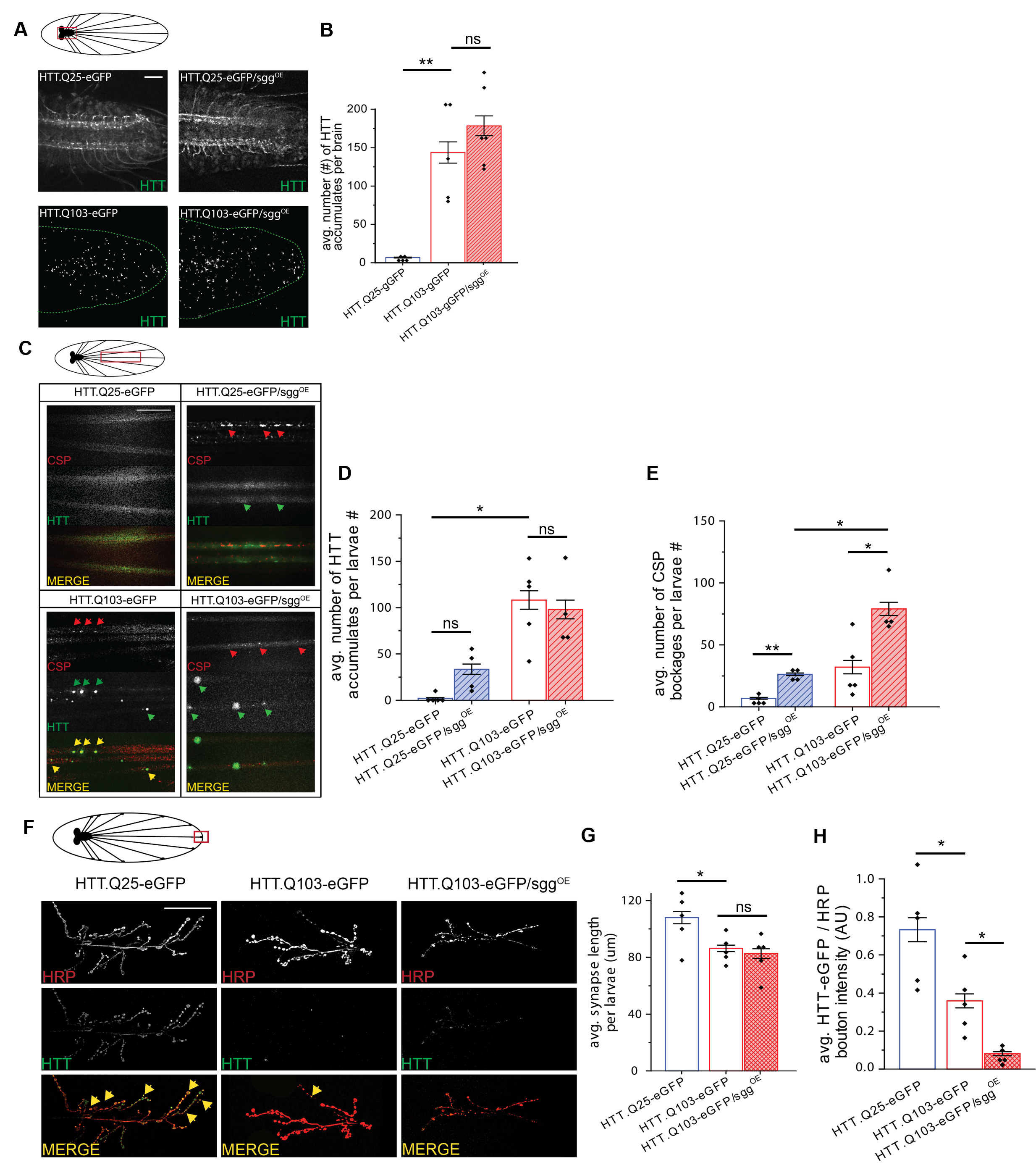

Supplement: Supplementary file 7 — Figure S7 [file 41419_2025_7524_MOESM7_ESM.png]
