# Supplementary material for: Opposing roles for GSK3β and ERK1-dependent phosphorylation of huntingtin during neuronal dysfunction and cell death in Huntington’s disease
Source: Cell Death Dis. 2025 Apr 22;16(1):328. doi: 10.1038/s41419-025-07524-0 (PMC12015319; doi:10.1038/s41419-025-07524-0)
Supplement: Supplementary file 8 — Table S1 [file 41419_2025_7524_MOESM8_ESM.docx]

| ProteinAC | Kinase | Description | TOTAL LMs | HTT-IP LMs |
| --- | --- | --- | --- | --- |
| Q12979 | ABR | ABR activator of RhoGEF and GTPase | LOST | -- |
| Q53H12 | AGK | acylglycerol kinase | LOST | -- |
| P27144 | AK4 | adenylate kinase 4 | LOST | -- |
| Q9Y6K8 | AK5 | adenylate kinase 5 | LOST | -- |
| P31751 | AKT2 | AKT serine/threonine kinase 2 | LOST | -- |
| Q13315 | ATM | ATM serine/threonine kinase | LOST | -- |
| O60885 | BRD4 | bromodomain containing 4 | LOST | -- |
| Q8IWQ3 | BRSK2 | BR serine/threonine kinase 2 | LOST | -- |
| Q16566 | CAMK4 | calcium/calmodulin dependent protein kinase IV | LOST | -- |
| Q9UQ88 | CDK11A | cyclin dependent kinase 11A | LOST | -- |
| Q9NYV4 | CDK12 | cyclin dependent kinase 12 | LOST | -- |
| Q00534 | CDK6 | cyclin dependent kinase 6 | LOST | -- |
| O14578 | CIT | citron rho-interacting serine/threonine kinase | LOST | -- |
| Q13057 | COASY | Coenzyme A synthase | LOST | -- |
| P48729 | CSNK1A1 | casein kinase 1 alpha 1 | LOST | -- |
| Q9Y4B6 | DCAF1 | DDB1 and CUL4 associated factor 1 | LOST | -- |
| Q8WVC6 | DCAKD | dephospho-CoA kinase domain containing | LOST | -- |
| P27707 | DCK | deoxycytidine kinase | LOST | -- |
| Q12959 | DLG1 | discs large MAGUK scaffold protein 1 | LOST | -- |
| P23919 | DTYMK | deoxythymidylate kinase | LOST | -- |
| Q15768 | EFNB3 | ephrin B3 | LOST | -- |
| Q9P2K8 | EIF2AK4 | eukaryotic translation initiation factor 2 alpha kinase 4 | LOST | -- |
| Q9HBU6 | ETNK1 | ethanolamine kinase 1 | LOST | -- |
| Q8N0W3 | FCSK | fucose kinase | LOST | -- |
| P51570 | GALK1 | galactokinase 1 | LOST | -- |
| Q9Y223 | GNE | glucosamine (UDP-N-acetyl)-2-epimerase/N-acetylmannosamine kinase | LOST | -- |
| P08069 | IGF1R | insulin like growth factor 1 receptor | LOST | -- |
| Q8IVT5 | KSR1 | kinase suppressor of ras 1 | LOST | -- |
| P53667 | LIMK1 | LIM domain kinase 1 | LOST | -- |
| Q9Y6R4 | MAP3K4 | mitogen-activated protein kinase kinase kinase 4 | LOST | -- |
| P53778 | MAPK12 | mitogen-activated protein kinase 12 | LOST | -- |
| Q16539 | MAPK14 | mitogen-activated protein kinase 14 | LOST | -- |
| Q13164 | MAPK7 | mitogen-activated protein kinase 7 | LOST | -- |
| P49137 | MAPKAPK2 | MAPK activated protein kinase 2 | LOST | -- |
| Q16644 | MAPKAPK3 | MAPK activated protein kinase 3 | LOST | -- |
| Q6P0Q8 | MAST2 | microtubule associated serine/threonine kinase 2 | LOST | -- |
| Q9UHY1 | NRBP1 | nuclear receptor binding protein 1 | LOST | -- |
| Q5VST9 | OBSCN | obscurin, cytoskeletal calmodulin and titin-interacting RhoGEF | LOST | -- |
| Q96RG2 | PASK | PAS domain containing serine/threonine kinase | LOST | -- |
| Q15118 | PDK1 | pyruvate dehydrogenase kinase 1 | LOST | -- |
| Q15120 | PDK3 | pyruvate dehydrogenase kinase 3 | LOST | -- |
| O15530 | PDPK1 | 3-phosphoinositide dependent protein kinase 1 | LOST | -- |
| O00443 | PIK3C2A | phosphatidylinositol-4-phosphate 3-kinase catalytic subunit type 2 alpha | LOST | -- |
| O00750 | PIK3C2B | phosphatidylinositol-4-phosphate 3-kinase catalytic subunit type 2 beta | LOST | -- |
| Q99755 | PIP5K1A | phosphatidylinositol-4-phosphate 5-kinase type 1 alpha | LOST | -- |
| O43314 | PPIP5K2 | diphosphoinositol pentakisphosphate kinase 2 | LOST | -- |
| Q9Y478 | PRKAB1 | protein kinase AMP-activated non-catalytic subunit beta 1 | LOST | -- |
| P10644 | PRKAR1A | protein kinase cAMP-dependent type I regulatory subunit alpha | LOST | -- |
| P41743 | PRKCI | protein kinase C iota | LOST | -- |
| Q9BZL6 | PRKD2 | protein kinase D2 | LOST | -- |
| Q13523 | PRPF4B | pre-mRNA processing factor 4B | LOST | -- |
| P50749 | RASSF2 | Ras association domain family member 2 | LOST | -- |
| Q15418 | RPS6KA1 | ribosomal protein S6 kinase A1 | LOST | -- |
| Q8IZE3 | SCYL3 | SCY1 like pseudokinase 3 | LOST | -- |
| Q9UHJ6 | SHPK | sedoheptulokinase | LOST | -- |
| Q15772 | SPEG | striated muscle enriched protein kinase | LOST | -- |
| Q96SB4 | SRPK1 | SRSF protein kinase 1 | LOST | -- |
| P78362 | SRPK2 | SRSF protein kinase 2 | LOST | -- |
| Q9P289 | STK26 | serine/threonine kinase 26 | LOST | -- |
| Q15750 | TAB1 | TGF-beta activated kinase 1 (MAP3K7) binding protein 1 | LOST | -- |
| Q96S44 | TP53RK | TP53 regulating kinase | LOST | -- |
| O15164 | TRIM24 | tripartite motif containing 24 | LOST | -- |
| Q9BZX2 | UCK2 | uridine-cytidine kinase 2 | LOST | -- |
| Q9ULX6 | AKAP8L | A-kinase anchoring protein 8 like | GAINED | -- |
| P22694 | PRKACB | protein kinase cAMP-activated catalytic subunit beta | GAINED | -- |
| P30085 | CMPK1 | cytidine/uridine monophosphate kinase 1 | SIG. INCREASED | -- |
| Q6PCE3 | PGM2L1 | phosphoglucomutase 2 like 1 | SIG. INCREASED | -- |
| P46527 | CDKN1B | cyclin dependent kinase inhibitor 1B | SIG. INCREASED | -- |
| P61024 | CKS1B | CDC28 protein kinase regulatory subunit 1B | INCREASED | -- |
| O75914 | PAK3 | p21 (RAC1) activated kinase 3 | INCREASED | -- |
| P19525 | EIF2AK2 | eukaryotic translation initiation factor 2 alpha kinase 2 | DECREASED | -- |
| P11717 | IGF2R | insulin like growth factor 2 receptor | DECREASED | -- |
| P53779 | MAPK10 | mitogen-activated protein kinase 10 | DECREASED | -- |
| Q8TD19 | NEK9 | NIMA related kinase 9 | DECREASED | -- |
| Q13464 | ROCK1 | Rho associated coiled-coil containing protein kinase 1 | DECREASED | -- |
| P51812 | RPS6KA3 | ribosomal protein S6 kinase A3 | DECREASED | -- |
| P11387 | TOP1 | DNA topoisomerase I | DECREASED | -- |
| Q9H4A3 | WNK1 | WNK lysine deficient protein kinase 1 | DECREASED | -- |
| P54886 | ALDH18A1 | aldehyde dehydrogenase 18 family member A1 | SIG. DECREASED | -- |
| P55263 | ADK | adenosine kinase | UNCHANGED | -- |
| P11274 | BCR | BCR activator of RhoGEF and GTPase | UNCHANGED | -- |
| Q9H0C8 | ILKAP | ILK associated serine/threonine phosphatase | UNCHANGED | -- |
| P52564 | MAP2K6 | mitogen-activated protein kinase kinase 6 | UNCHANGED | -- |
| O95819 | MAP4K4 | mitogen-activated protein kinase kinase kinase kinase 4 | UNCHANGED | -- |
| Q9UJ70 | NAGK | N-acetylglucosamine kinase | UNCHANGED | -- |
| O00764 | PDXK | pyridoxal kinase | UNCHANGED | -- |
| P07947 | YES1 | YES proto-oncogene 1, Src family tyrosine kinase | UNCHANGED | -- |
| P54819 | AK2 | adenylate kinase 2 | UNCHANGED | -- |
| Q7KZI7 | MARK2 | microtubule affinity regulating kinase 2 | UNCHANGED | -- |
| P31323 | PRKAR2B | protein kinase cAMP-dependent type II regulatory subunit beta | UNCHANGED | -- |
| Q8WZ42 | TTN | titin | UNCHANGED | -- |
| Q9UIJ7 | AK3 | adenylate kinase 3 | UNCHANGED | -- |
| O14936 | CASK | calcium/calmodulin dependent serine protein kinase | UNCHANGED | -- |
| P19784 | CSNK2A2 | casein kinase 2 alpha 2 | UNCHANGED | -- |
| P67870 | CSNK2B | casein kinase 2 beta | UNCHANGED | -- |
| Q8N568 | DCLK2 | doublecortin like kinase 2 | UNCHANGED | -- |
| Q16854 | DGUOK | deoxyguanosine kinase | UNCHANGED | -- |
| O14964 | HGS | hepatocyte growth factor-regulated tyrosine kinase substrate | UNCHANGED | -- |
| P19367 | HK1 | hexokinase 1 | UNCHANGED | -- |
| P27448 | MARK3 | microtubule affinity regulating kinase 3 | UNCHANGED | -- |
| Q03426 | MVK | mevalonate kinase | UNCHANGED | -- |
| O43252 | PAPSS1 | 3'-phosphoadenosine 5'-phosphosulfate synthase 1 | UNCHANGED | -- |
| P48426 | PIP4K2A | phosphatidylinositol-5-phosphate 4-kinase type 2 alpha | UNCHANGED | -- |
| P17612 | PRKACA | protein kinase cAMP-activated catalytic subunit alpha | UNCHANGED | -- |
| O75569 | PRKRA | protein activator of interferon induced protein kinase EIF2AK2 | UNCHANGED | -- |
