# Supplementary material for: Opposing roles for GSK3β and ERK1-dependent phosphorylation of huntingtin during neuronal dysfunction and cell death in Huntington’s disease
Source: Cell Death Dis. 2025 Apr 22;16(1):328. doi: 10.1038/s41419-025-07524-0 (PMC12015319; doi:10.1038/s41419-025-07524-0)
Supplement: Supplementary file 9 — Table S2 [file 41419_2025_7524_MOESM9_ESM.docx]

| ProteinAC | Kinase | Description | TOTAL LMs | HTT-IP LMs |
| --- | --- | --- | --- | --- |
| Q9Y243 | AKT3 | AKT serine/threonine kinase 3 | LOST | GAINED |
| Q8NCB2 | CAMKV | CaM kinase like vesicle associated | LOST | GAINED |
| O14976 | GAK | cyclin G associated kinase | LOST | GAINED |
| P52789 | HK2 | hexokinase 2 | LOST | GAINED |
| Q13418 | ILK | integrin linked kinase | LOST | GAINED |
| O60229 | KALRN | kalirin RhoGEF kinase | LOST | GAINED |
| P45985 | MAP2K4 | mitogen-activated protein kinase kinase 4 | LOST | GAINED |
| P42345 | MTOR | mechanistic target of rapamycin kinase | LOST | GAINED |
| Q4G0N4 | NADK2 | NAD kinase 2, mitochondrial | LOST | GAINED |
| Q99570 | PIK3R4 | phosphoinositide-3-kinase regulatory subunit 4 | LOST | GAINED |
| Q16512 | PKN1 | protein kinase N1 | LOST | GAINED |
| Q16513 | PKN2 | protein kinase N2 | LOST | GAINED |
| Q13131 | PRKAA1 | protein kinase AMP-activated catalytic subunit alpha 1 | LOST | GAINED |
| P54619 | PRKAG1 | protein kinase AMP-activated non-catalytic subunit gamma 1 | LOST | GAINED |
| Q96KG9 | SCYL1 | SCY1 like pseudokinase 1 | LOST | GAINED |
| Q6P3W7 | SCYL2 | SCY1 like pseudokinase 2 | LOST | GAINED |
| Q9UEW8 | STK39 | serine/threonine kinase 39 | LOST | GAINED |
| Q9Y5S2 | CDC42BPB | CDC42 binding protein kinase beta | SIG. DECREASED | GAINED |
| Q13308 | PTK7 | protein tyrosine kinase 7 (inactive) | SIG. DECREASED | GAINED |
| O75116 | ROCK2 | Rho associated coiled-coil containing protein kinase 2 | SIG. DECREASED | GAINED |
| Q3LXA3 | TKFC | triokinase and FMN cyclase | SIG. DECREASED | GAINED |
| Q12802 | AKAP13 | A-kinase anchoring protein 13 | DECREASED | GAINED |
| P31749 | AKT1 | AKT serine/threonine kinase 1 | DECREASED | GAINED |
| Q00536 | CDK16 | cyclin dependent kinase 16 | DECREASED | GAINED |
| P41240 | CSK | C-terminal Src kinase | DECREASED | GAINED |
| Q02750 | MAP2K1 | mitogen-activated protein kinase kinase 1 | DECREASED | GAINED |
| P27361 | MAPK3 | mitogen-activated protein kinase 3 | DECREASED | GAINED |
| Q2M2I8 | AAK1 | AP2 associated kinase 1 | INCREASED | GAINED |
| Q96SB3 | PPP1R9B | protein phosphatase 1 regulatory subunit 9B | INCREASED | GAINED |
| P29323 | EPHB2 | EPH receptor B2 | SIG. INCREASED | GAINED |
| P49841 | GSK3β | glycogen synthase kinase 3 beta | SIG. INCREASED | GAINED |
| P49840 | GSK3α | glycogen synthase kinase 3 alpha | SIG. INCREASED | GAINED |
| Q9HA64 | FN3KRP | fructosamine 3 kinase related protein | GAINED | GAINED |
| Q9NVE7 | PANK4 | pantothenate kinase 4 (inactive) | UNCHANGED | GAINED |
| Q96KB5 | PBK | PDZ binding kinase | UNCHANGED | GAINED |
| P11908 | PRPS2 | phosphoribosyl pyrophosphate synthetase 2 | UNCHANGED | GAINED |
| Q05397 | PTK2 | protein tyrosine kinase 2 | UNCHANGED | GAINED |
| P61925 | PKIA | cAMP-dependent protein kinase inhibitor alpha | UNCHANGED | GAINED |
| P04049 | RAF1 | Raf-1 proto-oncogene, serine/threonine kinase | UNCHANGED | GAINED |
| O75962 | TRIO | trio Rho guanine nucleotide exchange factor | UNCHANGED | GAINED |
| P24941 | CDK2 | cyclin dependent kinase 2 | UNCHANGED | GAINED |
| Q00535 | CDK5 | cyclin dependent kinase 5 | UNCHANGED | GAINED |
| Q9H479 | FN3K | fructosamine 3 kinase | UNCHANGED | GAINED |
| Q16774 | GUK1 | guanylate kinase 1 | UNCHANGED | GAINED |
| P36507 | MAP2K2 | mitogen-activated protein kinase kinase 2 | UNCHANGED | GAINED |
| P45983 | MAPK8 | mitogen-activated protein kinase 8 | UNCHANGED | GAINED |
| P78356 | PIP4K2B | phosphatidylinositol-5-phosphate 4-kinase type 2 beta | UNCHANGED | GAINED |
| Q15126 | PMVK | phosphomevalonate kinase | UNCHANGED | GAINED |
| Q9H2G2 | SLK | STE20 like kinase | UNCHANGED | GAINED |
| P12931 | SRC | SRC proto-oncogene, non-receptor tyrosine kinase | UNCHANGED | GAINED |
| Q13043 | STK4 | serine/threonine kinase 4 | UNCHANGED | GAINED |
| P13861 | PRKAR2A | protein kinase cAMP-dependent type II regulatory subunit alpha | SIG. INCREASED | SIG. INCREASED |
| P00568 | AK1 | adenylate kinase 1 | SIG. INCREASED | SIG. INCREASED |
| P06493 | CDK1 | cyclin dependent kinase 1 | UNCHANGED | SIG. INCREASED |
| Q13177 | PAK2 | p21 (RAC1) activated kinase 2 | UNCHANGED | SIG. INCREASED |
| Q13263 | TRIM28 | tripartite motif containing 28 | UNCHANGED | SIG. INCREASED |
| O15075 | DCLK1 | doublecortin like kinase 1 | SIG. INCREASED | INCREASED |
| P49903 | SEPHS1 | selenophosphate synthetase 1 | SIG. INCREASED | INCREASED |
| P17858 | PFKL | phosphofructokinase, liver type | UNCHANGED | INCREASED |
| O95747 | OXSR1 | oxidative stress responsive kinase 1 | SIG. DECREASED | DECREASED |
| P78527 | PRKDC | protein kinase, DNA-activated, catalytic subunit | UNCHANGED | DECREASED |
| P14618 | PKM | pyruvate kinase M1/2 | UNCHANGED | DECREASED |
| P12277 | CKB | creatine kinase B | UNCHANGED | DECREASED |
| P68400 | CSNK2A1 | casein kinase 2 alpha 1 | UNCHANGED | DECREASED |
| P08237 | PFKM | phosphofructokinase, muscle | UNCHANGED | DECREASED |
| P42356 | PI4KA | phosphatidylinositol 4-kinase alpha | UNCHANGED | LOST |
| Q01813 | PFKP | phosphofructokinase, platelet | DECREASED | UNCHANGED |
| Q13153 | PAK1 | p21 (RAC1) activated kinase 1 | INCREASED | UNCHANGED |
| O75131 | CPNE3 | copine 3 | UNCHANGED | UNCHANGED |
| P15531 | NME1 | NME/NM23 nucleoside diphosphate kinase 1 | UNCHANGED | UNCHANGED |
| P00558 | PGK1 | phosphoglycerate kinase 1 | UNCHANGED | UNCHANGED |
| P63104 | YWHAZ | tyrosine 3-monooxygenase/tryptophan 5-monooxygenase activation protein zeta | UNCHANGED | UNCHANGED |
| P0DP23 | CALM1 | calmodulin 1 | UNCHANGED | UNCHANGED |
| P27708 | CAD | carbamoyl-phosphate synthetase 2, aspartate transcarbamylase, and dihydroorotase | UNCHANGED | UNCHANGED |
| Q13557 | CAMK2D | calcium/calmodulin dependent protein kinase II delta | UNCHANGED | UNCHANGED |
| P28482 | MAPK1 | mitogen-activated protein kinase 1 | UNCHANGED | UNCHANGED |
| P22392 | NME2 | NME/NM23 nucleoside diphosphate kinase 2 | UNCHANGED | UNCHANGED |
| P60891 | PRPS1 | phosphoribosyl pyrophosphate synthetase 1 | UNCHANGED | UNCHANGED |
